# Exposure to Second-Hand Smoke and the Risk of Tuberculosis in Children and Adults: A Systematic Review and Meta-Analysis of 18 Observational Studies

**DOI:** 10.1371/journal.pmed.1001835

**Published:** 2015-06-02

**Authors:** Jayadeep Patra, Mehak Bhatia, Wilson Suraweera, Shaun K. Morris, Cyril Patra, Prakash C. Gupta, Prabhat Jha

**Affiliations:** 1 Centre for Global Health Research, St Michael’s Hospital, Toronto, Ontario, Canada; 2 Dalla-Lana School of Public Health, University of Toronto, Ontario, Toronto, Canada; 3 Division of Infectious Diseases, Hospital for Sick Children, Toronto, Ontario, Canada; 4 Department of Pediatrics, University of Toronto, Toronto, Ontario, Canada; 5 School of Public Health, University of Memphis, Memphis, Tennessee, United States of America; 6 Healis–Sekhsaria Institute for Public Health, Mumbai, India; McGill University, CANADA

## Abstract

**Background:**

According to WHO Global Health Estimates, tuberculosis (TB) is among the top ten causes of global mortality and ranks second after cardiovascular disease in most high-burden regions. In this systematic review and meta-analysis, we investigated the role of second-hand smoke (SHS) exposure as a risk factor for TB among children and adults.

**Methods and Findings:**

We performed a systematic literature search of PubMed, Embase, Scopus, Web of Science, and Google Scholar up to August 31, 2014. Our a priori inclusion criteria encompassed only original studies where latent TB infection (LTBI) and active TB disease were diagnosed microbiologically, clinically, histologically, or radiologically. Effect estimates were pooled using fixed- and random-effects models. We identified 18 eligible studies, with 30,757 children and 44,432 adult non-smokers, containing SHS exposure and TB outcome data for inclusion in the meta-analysis. Twelve studies assessed children and eight studies assessed adult non-smokers; two studies assessed both populations. Summary relative risk (RR) of LTBI associated with SHS exposure in children was similar to the overall effect size, with high heterogeneity (pooled RR 1.64, 95% CI 1.00–2.83). Children showed a more than 3-fold increased risk of SHS-associated active TB (pooled RR 3.41, 95% CI 1.81–6.45), which was higher than the risk in adults exposed to SHS (summary RR 1.32, 95% CI 1.04–1.68). Positive and significant exposure–response relationships were observed among children under 5 y (RR 5.88, 95% CI 2.09–16.54), children exposed to SHS through any parent (RR 4.20, 95% CI 1.92–9.20), and children living under the most crowded household conditions (RR 5.53, 95% CI 2.36–12.98). Associations for LTBI and active TB disease remained significant after adjustment for age, biomass fuel (BMF) use, and presence of a TB patient in the household, although the meta-analysis was limited to a subset of studies that adjusted for these variables. There was a loss of association with increased risk of LTBI (but not active TB) after adjustment for socioeconomic status (SES) and study quality. The major limitation of this analysis is the high heterogeneity in outcomes among studies of pediatric cases of LTBI and TB disease.

**Conclusions:**

We found that SHS exposure is associated with an increase in the relative risk of LTBI and active TB after controlling for age, BMF use, and contact with a TB patient, and there was no significant association of SHS exposure with LTBI after adjustment for SES and study quality. Given the high heterogeneity among the primary studies, our analysis may not show sufficient evidence to confirm an association. In addition, considering that the TB burden is highest in countries with increasing SHS exposure, it is important to confirm these results with higher quality studies. Research in this area may have important implications for TB and tobacco control programs, especially for children in settings with high SHS exposure and TB burden.

## Abstract 2

In a systematic review and meta-analysis, Jayadeep Patra and colleagues explore associations between second-hand smoke exposure, demographics, lifestyle factors, and tuberculosis.

## Editors' Summary

**Background:**

Tuberculosis (TB)—a bacterial disease that usually affects the lungs—is a global public health problem. Every year, 8.6 million people develop active TB and at least 1.3 million people die from the disease, mainly in resource-limited countries. *Mycobacterium tuberculosis*, the organism that causes tuberculosis, is spread in airborne droplets when people with active TB cough or sneeze. If another person inhales these droplets, he or she may become infected. However, only about 10% of people who become infected develop active TB, the symptoms of which include cough, weight loss, and fever. Most infected individuals contain and control the infection. Indeed, it is thought that about one-third of the world’s population has latent TB infection (LTBI). Individuals with LTBI have no symptoms and are not infectious, but they may develop active disease later in life. Both LTBI and active TB can be cured by taking antibiotics daily for several months. LTBI needs to be treated to prevent the infection progressing to active disease; active TB needs to be treated to prevent death and the spread of tuberculosis.

**Why Was This Study Done?:**

Very young and very old people, and people who are immunocompromised because of, for example, infection with HIV, are at high risk of developing active TB. Other risk factors for contracting TB include living in crowded, unsanitary conditions and poor nutrition. Another possible risk factor for TB is smoking tobacco products. Tobacco smoke is thought to damage both the fine hairs lining the lungs that normally provide a defense against bacterial infections and the lungs’ immune system. But is exposure to second-hand smoke (SHS; passive smoking) also a risk factor for TB? It is particularly important to know whether there is an association between exposure to SHS and TB among children because in some countries with a high TB burden a large proportion of children are exposed to SHS. In this systematic review (a study that uses predefined criteria to identify all the research on a given topic) and meta-analysis (a statistical approach for combining the results of several studies), the researchers investigate whether SHS exposure is a risk factor for TB among children and adults.

**What Did the Researchers Do and Find?:**

The researchers identified 18 observational studies that recorded diagnoses of LTBI or active TB, exposure to SHS, and exposure to some other risk factors for TB among 30,757 children and 44,432 adult non-smokers. Compared to children not exposed to SHS, exposed children had a pooled relative risk (RR) of LTBI of 1.64. That is, exposure to SHS among children was associated with a 1.64-fold increased risk of LTBI. SHS exposure was also associated with a 3.41-fold increased risk of active TB among children and with a 1.32-fold increased risk of active TB among adults. Other analyses indicated that although there was an association between exposure to SHS and active TB in every age group, the highest relative risk was among children aged 0–5 years, and that children exposed to SHS living in crowded households had a higher relative risk of developing active TB than SHS-exposed children living in less crowded households. Importantly, the associations between SHS exposure and LTBI and active TB remained significant (unlikely to have occurred by chance) after adjustment for exposure to other known TB risk factors such as the presence of an individual with TB in the household.

**What Do These Findings Mean?:**

These findings suggest that exposure to SHS is associated with LTBI and active TB among children and adults after adjustment for a limited number of other risk factors for TB. Because the studies included in this meta-analysis were observational studies, individuals exposed to SHS might have also been exposed to other unknown factors (confounding factors) that were actually responsible for their increased risk of TB. Thus, these findings do not prove a causal link between exposure to SHS and TB. Moreover, because of high heterogeneity (variability) among the studies included in this meta-analysis (particularly those that included children), these findings need to be confirmed in additional studies. Such studies are essential because a causal link between SHS exposure and TB, if proved, will have important implications for the health of children living in countries with high SHS exposure and a high TB burden, and for the design of future TB and tobacco control programs.

**Additional Information:**

Please access these websites via the online version of this summary at http://dx.doi.org/10.1371/journal.pmed.1001835.

- The World Health Organization (WHO) provides information (in several languages) on tuberculosis; the *Global Tuberculosis Report 2014* provides information about tuberculosis around the world; WHO also provides information about the health risks associated with tobacco use (in several languages)
- The Stop TB Partnership is working towards tuberculosis elimination and provides personal stories about tuberculosis (in English and Spanish); the Tuberculosis Vaccine Initiative (a not-for-profit organization) also provides personal stories about tuberculosis
- The US Centers for Disease Control and Prevention provides information about tuberculosis (in English and Spanish), about smoking and tobacco use, and about the health effects of second-hand smoke
- The US National Institute of Allergy and Infectious Diseases also has detailed information on all aspects of tuberculosis
- The US Environmental Protection Agency provides information about the health effects of exposure to second-hand smoke in children
- MedlinePlus has links to further information about tuberculosis, about smoking, and about the effects of second-hand smoke (in English and Spanish)

## Introduction

According to recent global health estimates, tuberculosis (TB) is among the top ten causes of global mortality and ranks second after cardiovascular disease (CVD) in most high-burden regions [1,2]. In the latest WHO Global Tuberculosis Report [3], it was estimated that approximately one-third of the world’s population is infected with *Mycobacterium tuberculosis* (Mtb), and that each year 8.6 million people develop TB disease and 1.3 million die of the disease. Children contribute a substantial burden of new TB cases (6%) and deaths (8%) globally [3,4]. The international community of TB researchers has recognized the urgent need to address pediatric TB, and is now working toward reducing child deaths from TB to zero [5].

Among the many risk factors for TB, smoking of tobacco products has recently been restudied for its association with TB [6,7]. Biologically plausible mechanisms for the effect of smoking on risk of TB infection include decrease in ciliary function, alterations in macrophage number and response [8,9], and decrease in CD4 and CD8 cells that produce interferon gamma and TNF alpha [9]. There is some evidence indicating damaging effects of second-hand smoke (SHS; also referred to as involuntary smoking, passive smoking, or environmental tobacco smoke [ETS]) on the innate immune system of the lungs as well as the adaptive immune response, leading to a greater susceptibility to Mtb infection and progression to active TB [10,11]. However, the link between SHS exposure and TB is not fully understood. In an attempt to fill this gap, we conducted a systematic review and meta-analysis to examine the association of SHS exposure with the risk of latent TB infection (LTBI) and active TB disease in children and adult non-smokers.

## Methods

### Search Strategy

We did a systematic review (according to PRISMA guidelines) to identify studies related to SHS exposure and TB. We searched Medline (via PubMed) and Embase for studies published between January 1, 1946, and August 31, 2014 (for search terms see Box 1). Articles resulting from these searches and relevant references cited in those articles were reviewed.

**Box 1. Search Strategy and Terms Used to Identify Studies on Second-Hand Smoke Exposure and TB**

Medline term search:

1. “tuberculosis”
2. “second-hand smoking”
3. “environmental exposure”
4. “passive smoking”
5. “tobacco pollution”
6. “cohort studies” OR “case-control studies” OR “cross-sectional studies” OR “epidemiologic studies” OR “prospective studies” OR “ratio” OR “risk”
7. “(1) AND (2) AND (6)” OR “(1) AND (3) AND (6)” OR “(1) AND (4) AND (6)” OR “(1) AND (5) AND (6)”

Embase term search:

1. “tuberculosis”
2. “second-hand smoking”
3. “environmental exposure”
4. “passive smoking”
5. “tobacco pollution”
6. “cohort studies” OR “case-control studies” OR “cross-sectional studies” OR “epidemiologic studies” OR “prospective studies” OR “ratio” OR “risk”
7. “(8) AND (9) AND (13)” OR “(8) AND (10) AND (13)” OR “(8) AND (11) AND (13)” OR “(8) AND (12) AND (13)”

Google Scholar term search:

1. “tuberculosis”
2. “second-hand smoking”
3. “environmental exposure”
4. “passive smoking”
5. “tobacco pollution”
6. “cohort studies” OR “case-control studies” OR “cross-sectional studies” OR “epidemiologic studies” OR “prospective studies” OR “ratio” OR “risk”
7. “(15) AND (16) AND (20)” OR “(15) AND (17) AND (20)” OR “(15) AND (18) AND (20)” OR “(15) AND (19) AND (20)”

The searches and studies included were not limited by publication date, country, or language. Database searches were conducted independently by two authors (J. P. and M. B.). To ensure comprehensive acquisition of literature, independent supplemental manual searches were performed on the reference lists of relevant articles and other databases, including Scopus, Web of Science, and Google Scholar. The contents of the abstracts or full-text articles identified during the literature search were reviewed independently by two reviewers (J. P. and M. B.) to determine whether they met the criteria for inclusion.

### Inclusion and Exclusion

**Fig 1:**
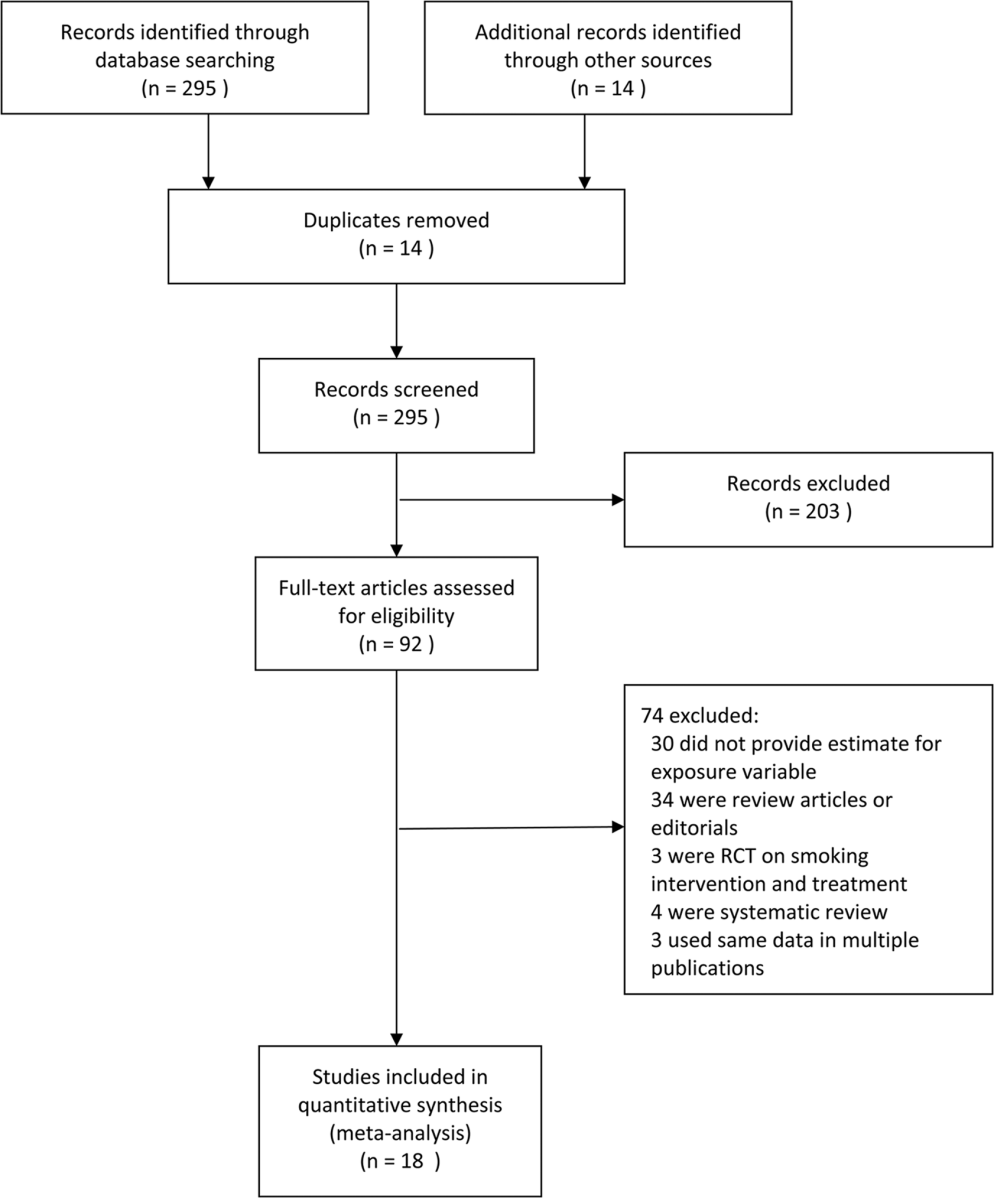
Flowchart of study identification and inclusion. RCT, randomized controlled trial.

Articles were independently identified by two reviewers for inclusion and in-depth examination. Our inclusion criteria selected only (1) original studies where LTBI or active TB were diagnosed with clinical, radiological, histological, and/or microbiological diagnostic tools; (2) cohort studies, case-control studies, or cross-sectional surveys (case reports, review articles, and editorials were excluded); and (3) studies on human participants aged ≤15 y, (i.e., children) or >15 y (i.e., adults). For included articles reporting on the same study population, the more detailed article was selected. Discrepancies in article inclusion between reviewers were resolved by consensus. We categorized exposures as SHS or ETS exposure, parental smoking, household smoking or presence of household smoker(s), and regular contact with smokers. Study selection is summarized in Fig 1. The exclusion criteria disallowed studies in which active smoking was not distinguished from passive smoking, active smoking was the only exposure, or prenatal exposure was included. We excluded studies that explored high-risk populations with HIV or other immune-compromising conditions.

### Data Extraction and Synthesis

Data extracted included study location, setting (e.g., community, hospital, etc.), participant age, number of participants, exposure definition and levels, diagnosis of outcome, crude and adjusted effect sizes, and 95% confidence intervals (CIs). The presence of appropriate controls and the covariates used for adjustment in multivariate analysis were extracted as study design quality indicators. Study authors were contacted as necessary to obtain pertinent data not published in the articles. When necessary, we manually calculated the unadjusted odds ratio from raw data in an article for inclusion in the meta-analysis, or excluded the article if this was not possible. We assessed the quality of the included studies using the Newcastle–Ottawa scale (S1 Table) [12].

### Statistical Analysis

We conducted meta-analyses of risk estimates for LTBI and active TB disease for exposure to SHS compared with non-exposure to SHS in non-smoking children and adults, and we report pooled estimates and 95% CIs. For data extraction, analysis, and reporting, we used the PRISMA guidelines for meta-analysis of observational studies (S1 Text) [13]. We tested for and quantified heterogeneity with the Cochran Q statistic (*p* < 0.05) and *I* ^2^ statistic, respectively, to describe the variation in effect size attributable to heterogeneity across studies [14,15]. We used the *I* ^2^ statistic to select the pooling method: fixed-effects models for *I* ^2^ < 50% and random-effects models for *I* ^2^ ≥ 50% [14,15]. CIs of *I* ^2^ were calculated by the method suggested by Higgins and Thompson [15]. We summarized pooled odds ratios using the inverse-variance method for fixed-effects models and the DerSimonian and Laird method for random-effects models [14]. We used Galbraith plots to visualize the impact of individual studies on the overall homogeneity test statistic [16]. We used meta-regression to evaluate whether effect size estimates were significantly different by specific study characteristics and quality factors, specifically, adjustment for covariates and for laboratory-confirmed TB diagnosis (most rigorous method) versus a mix of clinical and laboratory-confirmed diagnosis (less rigorous). We also re-estimated the effect size stratified on the same study characteristics and quality factors to produce separate estimates. Subgroup analysis was conducted on all relevant study characteristics regardless of statistical significance. We investigated the presence and the effect of publication bias using a combination of the Begg’s test [15] and Egger’s test [17]. Statistical analyses were performed using Stata 12.1 (StataCorp). The metan, heterogi, metareg, metabias, galbr, metatrim, and metafunnel macros were used for meta-analytic procedures. *p*-Values < 0.05 were considered statistically significant. An overview of the study protocol is provided in S1 Protocol.

## Results

Our search strategy resulted in 309 studies, of which 92 were deemed relevant upon initial inspection of study titles. Eighteen studies, with 30,757 children and 44,432 adult non-smokers, met all of the inclusion criteria and were included in the meta-analysis (Fig 1). There was only one non-English (Spanish) study included in the analysis [18]. Six studies [19–24] had LTBI as an outcome of interest, and the remaining 12 studies [18,25–35] had active TB as an outcome of interest. Twelve studies [18–22,24,26,29,31,33–35] assessed children and eight studies [22,23,25,27,28,30–32] assessed adult non-smokers; two studies [22,31] assessed both populations. All studies with active TB as an outcome had information on dose–response by a variety of variables, including age [18,25–35], amount of smoke exposure in the household [21,26,33], contact with a TB patient [20,21,24,26,35], and household crowdedness [18,26,35], with at least three exposure categories. Table 1 summarizes the study characteristics. Given the significant heterogeneity among the effect estimates of these studies, we conducted sensitivity analyses with important study characteristics for each outcome for both children and adults.

**Table 1:** Study characteristics.

Category	Study Design	Author and Year, Country [Reference]	Setting, Population, and Mean Age	Exposure; Exposure Measurement	Outcome Measurement	Adjustment in Multivariate Analysis	Findings
**LTBI in children—pulmonary**	Cross-sectional	Babayigit-Hocaoglu et al. 2011, Turkey [19]	Hospital-based sample of 81 patients, including 35 with exposure to SHS; mean age = 8.5 y	Exposure to cigarette smoke in the household; patient records	TST+ (≥15 mm)	Only unadjusted ORs reported	Exposure to cigarette smoke showed a lower risk of LTBI than no such exposure
	Cross-sectional	den Boon et al. 2007, South Africa [20]	Population-based sample of 1,811 children (including 356 who previously lived with a TB patient); childhood TB prevalence 18%; mean age = 7.5 y	Exposure to ever-smoker in the household with smoking history of at least 1 y (and living with TB patient in the household); household members’ self-report on comprehensive lung-health survey	TST+ (≥10 mm)	Age, income of household members, presence of a patient with TB in the house	Exposure to ever smoker (and contact with a TB patient) in the household was related to higher risk of LTBI; risk was higher than exposure to household smoke alone
	Cross-sectional	Du Preez et al. 2011, South Africa [21]	Community-based sample of 324 children from three geographically adjacent, low-income urban communities; mean age = 6.8 y	Exposure to current smokers in the household; interviews with parents or primary caregivers	TST+ (≥10 mm)	Age, previous TB treatment, ethnicity, housing type/SES ratio of adults to children, presence of a patient with TB in the house, BMI, BMF	Exposure to two or more smokers in the household associated with LTBI
	Cross-sectional	Lindsay et al. 2014, US [22]	Nationally representative sample of 2,534 US-born and 408 foreign-born children, including 22 US-born and 36 foreign-born cases; mean age = 11.6 y	Exposure to smoker in the household; interviews with household members	TST+ (≥10 mm)	Age, gender, poverty income ratio, education, race, household size, having lived with someone with TB	Exposure to household smoke showed higher risk of LTBI only for foreign-born children
**LTBI in children—pulmonary or extrapulmonary**	Cross-sectional	Singh et al. 2005, India [24]	Hospital-based sample of 376 children; mean age = 3 y	Smoking status of adult contacts with TB; structured interviews with contacts	TST+ (≥10 mm)	Age, BCG vaccination, malnutrition, smear status of contact	Exposure to smoking by parents with TB associated with LTBI
**TB disease in children—pulmonary**	Cohort	Lin et al. 2013, Taiwan [31]	Population-based sample of 23,827 children from the NHIS; excluded those under 12 y; mean age = 15 y	Exposure to SHS in the household; household members’ self-report on NHIS	Mtb culture positivity; clinical evidence; CXR	Age, survey year, sex, education, marital status, residing in a crowded home, alcohol use, employment status, household income	Exposure to secondhand smoke in the household strongly associated with TB disease
	Case-control	Altet 1996, Spain [26]	Sample of 188 children, including 93 cases (exposed to a TB patient in the household) and 95 controls (close contacts without evidenceof active TB); mean age = 7.5 y	Exposure to tobacco smoked by others in the household at the time of survey and 6 mo prior; cotinine levels and parents/caregivers’ self-report on structured questionnaires	AFB smear and culture positivity; clinical and radiological evidence; TST+	Age, sex, father’s SES	Exposure to tobacco smoke in the household strongly associated with TB disease in children
** **	Case-control	García-Sancho et al. 2013, Mexico [18]	Hospital-based sample of 282 children, including 65 cases and 217 controls (non-smoking and with diseases of ear or mastoid process attending the same hospital); mean age = 6.2 y	Exposure to SHS in the household; parents/caregivers’ self-report on standardized questionnaire	Bacteriological evidence	Only unadjusted ORs reported	Exposure to passive tobacco smoke in the household associated with TB disease
	Case-control	Jubulis et al. 2014, India [29]	Hospital-based sample of 25 cases (definite and probable) and 118 healthy controls attending immunization clinics; primarily poor population; mean age = 3 y	Exposure to tobacco smoke in the household; household members’ self-report in interviews	Mtb culture positivity; clinical evidence; CXR; response to antibiotics	Age, SES, BMI, BMF, IAP, household TB exposure (contact)	Exposure to tobacco smoke in the household associated with TB disease
**TB disease in children—pulmonary or extrapulmonary**	Case-control	Patra et al. 2012, India [33]	Hospital-based sample of 400 children, including 200 cases and 200 age/sex-matched controls from the outpatient department of a public hospital from the same region; mean age = 10.34 y	Regular exposure to tobacco smoke in the household at the time of symptom appearance; parents’ self-report in structured interview	Diagnosis per standard WHO criteria (sputum smear and culture positivity)	Mother’s education, passive smoking, contact with a family member with TB diagnosed in the previous 2 y	Pulmonary TB was present in 47.5% of the cases; exposure to tobacco smoke in the household associated with TB disease
	Case-control	Ramachandran et al. 2011, India [34]	RNTCP-based sample of 123 children, including 41 cases and 82 controls from patient’s neighborhood; mean age = 5.94 y	Exposure to SHS or sidestream smoke from cigarette, cigar, or pipe; household members’ response to structured questionnaire	Diagnosis per standard WHO criteria (sputum smear and culture positivity)	Age, gender, income	Most cases (78%) had pulomonary TB; strong association between secondhand/side stream smoke and TB disease, both pulmonary and extrapulmonary
	Case-control	Tipayamongkholgul et al. 2005, Thailand [35]	Hospital-based sample of 260 children, including 130 cases and 130 age/sex-matched controls; mean age = 7.5 y	Exposure to passive smoke and contact with a TB patient in the household; self-report and medical records	Self-report by parents or guardians; medical records	Age, average number of persons/room, frequency of illness	Pulmonary TB comprised 50% of the cases; strong association between passive smoking and TB disease even in the absence of direct contact with a TB patient in the household
**LTBI in adults—pulmonary**	Cross-sectional	Lindsay et al. 2014, US [22]	Nationally representative sample of 2,664 US-born and 931 foreign-born adults, including 114 US-born and 167 foreign-born cases; mean age = 48.7 y	Passive smoke exposure in the household; self-report and serum cotinine levels (≥0.05 ng/ml)	TST+ (≥10 mm for non-vaccinated and ≥15 mm for BCG-vaccinated adults)	Age, gender, poverty income ratio, education, race, household size, having lived with someone with TB	Passive smoke associated with LTBI; risk higher in non-US-born adults
** **	Cross-sectional	Shin et al. 2013, Mexico [23]	Community-based sample of 262 injection drug users, including 28 cases; mean age = 38 y	Passive smoke exposure in the household; serum cotinine levels (10–30 ng/ml)	QFT-GIT+ (>0.35 IU/ml)	Age, sex, education, unstable housing, average monthly income, HIV infection	Association between passive smoke exposure and LTBI
**TB disease in adults—pulmonary**	Cohort	Lin et al. 2013, Taiwan [31]	Nationally representative sample of 23,827 non-smoking adults, including 85 cases; mean age = 38.5 y	Exposure to SHS in the household; household members’ self-report on NHIS	Bacteriological confirmation or clinical evidence of signs/symptoms; CXR; response to antibiotics	Age, survey year, sex, education, marital status, residing in a crowded home, alcohol use, employment status, household income	Association between household secondhand smoke exposure; risk decreased with increasing age, with a protective effect in those above 60y
** **	Case-control	Alcaide et al. 1996, Spain [25]	Hospital-based sample of 92 adults, including 46 cases and 46 controls recruited from close contacts of cases; mean age = 20 y	Exposure to tobacco smoked by others; self-report and cotinine concentration in 24-h urine samples	AFB culture positivity on sputum, bronchial, or bronchoalveolar specimen or clinical, radiological, and skin testing (TST ≥ 5 mm)	Age, gender, SES, household TB exposure	Exposure to tobacco smoke by others associated with TB disease
	Case-control	Ariyothai et al. 2004, Thailand [27]	Hospital-based sample of 100 cases and 100 age/sex-matched controls (non-TB patients or healthy individuals); mean age = 33.28 y	Exposure to SHS at home, work, or public places; both currently and ≥6 mo prior to the interview; self-report in structured interview	Sputum AFB smear positivity; CXR	Age, sex, BMI	Association between second hand smoke exposure and TB disease
** **	Case-control	Gupta et al. 2013, India [28]	Hospital-based sample of 100 cases and 100 healthy controls; mean age = 39.50 y	Exposure to passive smoking by household members; self-report in structured interview	Sputum AFB smear and/or culture positivity	Age, sex, education, SES, religion, cooking fuel used	Association between ETS and TB disease
	Case-control	Oztürk et al. 2014, Turkey [32]	Hospital-based sample of 362 cases and 408 age-matched controls; mean age = 42.5 y	Never-smoker exposed to ETS at home; self-report in structured interview	Sputum AFB smear and/or culture positivity	Only unadjusted ORs reported	ETS exposure associated with TB disease
**TB disease in adults—pulmonary or extrapulmonary**	Cohort	Leung et al. 2010, Hong Kong [30]	Community-based sample of 15,486 never-smoking women including 117 cases, recruited from 18 elderly health centers; mean age = 69.5 y	Exposure to one or more smokers living in the same household; database of Elderly Health Service	Bacteriological confirmation or clinical, radiological, and/or histological evidence	Age, Cantonese speaking, education, housing, alcohol use, obstructive lung disease, hypertension, heart disease, CVD, diabetes mellitus	Exposure to smokers in the household associated with TB disease

AFB, acid-fast bacilli; BCG, Bacillus Calmette–Guérin; BMF, biomass fuel; BMI, biomass index; CXR, chest, X-ray; IAP, indoor air pollution; IU, international units; NHIS, National Health Interview Survey; OR, odds ratio; QFT-GIT, QuantiFERON TB Gold In-Tube test; RNTCP, Revised National Tuberculosis Control Programme; SES, socioeconomic status; TST, tuberculin skin test.

### Second-Hand Smoke Exposure and Latent TB Infection

**Fig 2:**
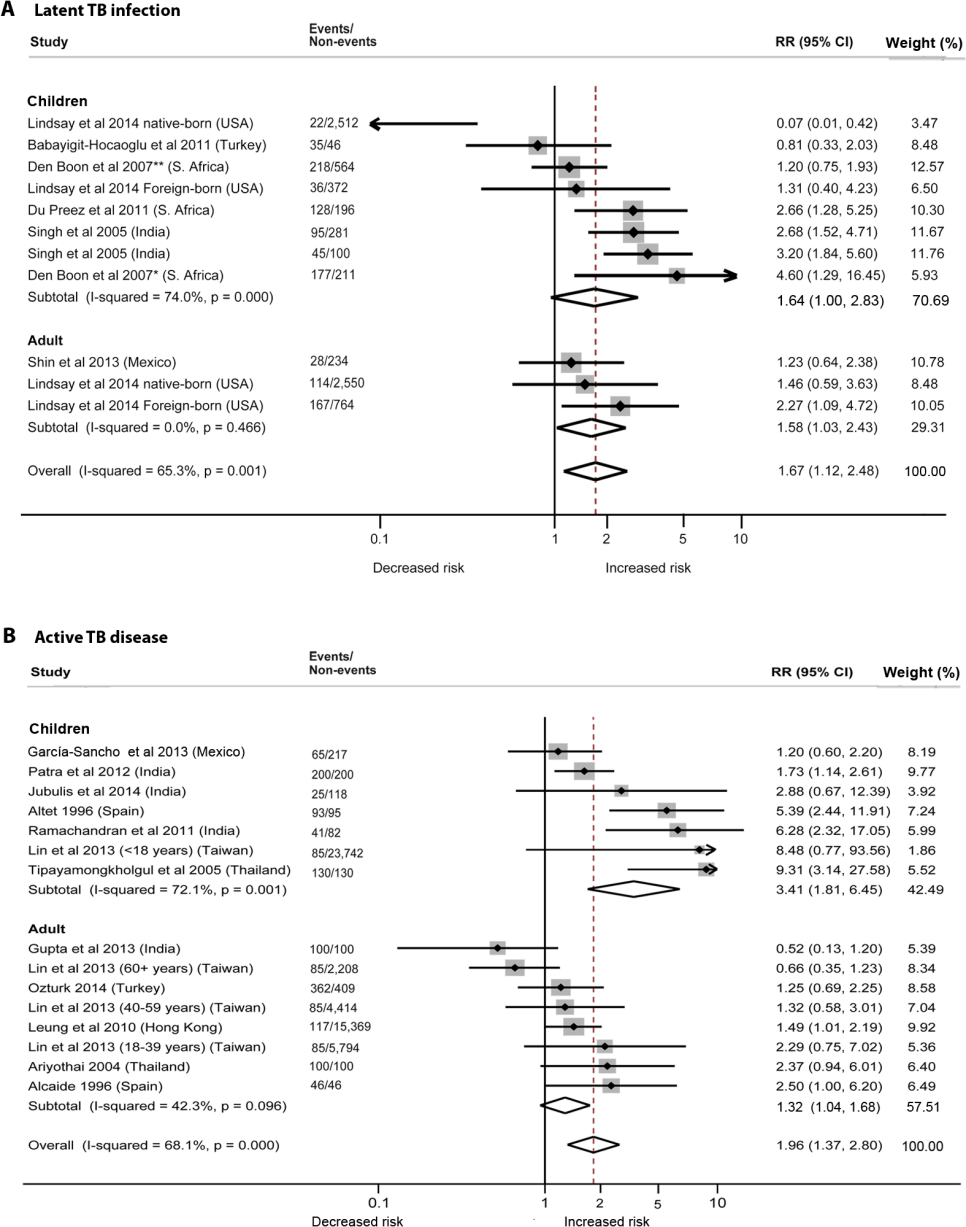
Risk of latent TB infection and active TB disease for second-hand smoke exposure compared to non-exposure in children and adults. (A) LTBI; (B) active TB disease. Singh et al. [24] reported SHS risks for children with contacts with sputum-negative (95/281) and sputum-positive TB patients (45/100). The effect estimate (diamond) for US-born children in the study by Lindsay et al. [22] is not displayed due to its smaller size and weight. Lin et al. [31] did not report age-stratified TB cases. Weights are from random-effects analysis. *Patient with TB living in house. **No patient with TB living in house.

We identified six studies [19–24] reporting the relative risk (RR) of LTBI upon exposure to SHS (Table 2). All six studies were cross-sectional, and half [20–22] were assessed to be of good quality. These studies were conducted in diverse WHO regions including Europe [19], South-East Asia [24], Africa [20,21], and the Americas [22,23]. Overall, LTBI was positively associated with SHS exposure (pooled RR 1.67, 95% CI 1.12–2.48, *p* for heterogeneity = 0.001, *I* ^2^ = 65.3%) (see Fig 2A). Relative risk was higher in the South-East Asia Region (RR 2.93, 95% CI 1.97–4.36) and African Region (RR 2.11, 95% CI 1.00–4.49) than in the other regions. Our analysis was limited since only a few studies adjusted for potentially confounding variables. However, among the studies that did show adjusted estimates, effects were significant after adjustment for age (RR 1.78, 95% CI 1.19–2.68), biomass fuel (BMF) use (RR 2.66, 95% CI 1.31–5.39), and contact with a TB patient in the household (RR 2.03, 95% CI 1.25–3.28) (Table 2). There was no significant association between SHS exposure and LTBI after a combined adjustment for age and socioeconomic status (SES) (RR 1.11, 95% CI 0.54–2.31) or after adjustment for just SES (RR 1.50, 95% CI 0.93–2.43), although age- and SES-adjusted estimates were significant in the subgroup analysis for adults (pooled RR 1.58, 95% CI 1.03–2.43, *p* for heterogeneity = 0.466, *I* ^2^ = 0.0%) (S2 Table) [22,23]. Meta-regression analysis showed significant effect size modification for studies adjusted for SES (*p =* 0.033) and marginal significance for studies adjusted for presence of a TB patient in the household (*p =* 0.105).

**Table 2:** Quality assessment and subgroup analysis: second-hand smoke exposure and latent TB infection.

Measure or Outcome	Study Characteristic (Number of Studies)	Summary Estimate	95% CI	*I* ^*2*^ (95% CI)	Meta-Regression	
					Coefficient (95% CI)	*p*-Value
Population*	All studies (6) [19–24]	1.67	1.12–2.48	65.3% (34.1%–81.7%)	(..)	(..)
	Children (5) [19–22,24]	1.64	1.00–2.83	74.0% (47.2%–87.2%)	0.05 (−1.25, 1.35)	0.933
	Adults (2) [22,23]	1.58	1.03–2.43	0.0% (0.0%–89.6%)	Ref	
**Quality of study**	Good (3) [20–22]	1.53	0.86–2.73	66.4% (24.9%–85.0%)	−0.16 (−1.35, 1.03)	0.770
	Poor to moderate (3) [19,23,24]	1.83	1.02–3.29	68.9% (10.2%–89.3%)	Ref	
**WHO region**	EUR (1) [19]	0.81	0.33–2.01	(..)	0.52 (−0.81, 1.85)	0.387
	SEAR (1) [24]	2.93	1.97–4.36	0.0%	0.88 (−0.52, 2.28)	0.180
	AFR (2) [20,21]	2.11	1.00–4.49	66.6% (0.0%–90.4%)	−0.40 (−2.49, 1.68)	0.662
	AMR (2) [22,23]	1.11	0.54–2.31	65.7% (10.5%–86.9%)	Ref	
**Multivariate adjusted analysis**	Yes (5) [20–24]	1.78	1.19–2.68	65.0% (31.3%–82.2%)	0.80 (−1.13, 2.73)	0.372
	No (1) [19]	0.81	0.33–2.01	(..)	Ref	
**Adjusted for age**	Yes (5) [20–24]	1.78	1.19–2.68	65.0% (31.3%–82.2%)	0.80 (−1.13, 2.73)	0.372
	No (1) [19]	0.81	0.33–2.01	(..)	Ref	
**Adjusted for SES**	Yes (4) [20–23]	1.50	0.93–2.43	61.8% (17.4%–82.3%)	−0.78 (−1.49, −0.08)	0.033
	No (2) [19,24]	2.09	1.04–4.17	70.0% (0.0%–91.2%)	Ref	
**Adjusted for age and SES**	Yes (2) [22,23]	1.11	0.54–2.31	65.7% (10.5%–86.9%)	−0.57 (−1.67, 0.52)	0.268
	No (4) [19–21,24]	2.09	1.30–3.34	65.1% (16.1%–85.5%)	Ref	
**Adjusted for cooking/BMF**	Yes (1) [21]	2.66	1.31–5.39	(..)	0.52 (−1.35, 2.40)	0.544
	No (5) [19,20,22–24]	1.57	1.02–2.42	67.2% (36.3%–83.1%)	Ref	
**TB contact in the household**	Yes (4) [20–22,24]	2.03	1.25–3.28	61.5% (16.6%–82.2%)	0.66 (−0.17, 1.50)	0.105
	No (3) [19,20,23]	1.21	0.82–1.78	0.0%	Ref	
**Adjusted for TB contact in the household**	Yes (4) [20–22,24]	2.03	1.25–3.28	61.5% (16.6%–82.2%)	(..)	(..)
**Mode of diagnosis**	TST/QFT (6) [19–24]	1.67	1.12–2.48	65.3% (34.1%–81.7%)	(..)	(..)

*The overall pooled RR after removing an outlier (US-born children in [22]) was 1.87 (1.50–2.32), with a slightly decreased heterogeneity (*I* ^2^ = 47.4%, 95% CI 0.0%–74.7%, *p* = 0.047). “(..)” indicates that the nominated reference group was not available to compute meta-regression statistics.

AFR, African Region; AMR, Region of the Americas; EUR, European Region; QFT-GIT, QuantiFERON TB Gold In-Tube test; SEAR, South-East Asia Region; TST, tuberculin skin test.

The summary relative risk of LTBI associated with SHS exposure in children derived from five studies [19–22,24] was similar to the overall effect size (pooled RR 1.64, 95% CI 1.00–2.83), but substantial heterogeneity was observed (*I* ^2^ = 74.0%). Both the Galbraith plot and funnel plot showed that one study estimate from US [22] was a potential source of heterogeneity. The distinct findings (very low effect size) in this study may have resulted from a difference in the methodology: stratification of the sample in the pediatric population by their birth place (US-born versus foreign-born). When this single study estimate (RR = 0.07) was excluded, the overall effect estimate increased and the heterogeneity score improved (pooled RR 1.87, 95% CI 1.50–2.32, *p* for heterogeneity = 0.047, *I* ^2^ = 47.4%); therefore, in a subsequent LTBI analysis for children we presented a sensitivity analysis without the outlier (see S2 Table for pooled and subgroup analysis). With this outlier removed, summary relative risk of LTBI associated with SHS exposure in children was higher than the overall effect size, with modest heterogeneity (pooled RR 2.00, 95% CI 1.30–3.08, *p* for heterogeneity = 0.022, *I* ^2^ = 49.5%) (S2 Table). Three [20,21,24] of the five studies in children reported a positive and significant association between SHS exposure and LTBI (Fig 2A). Four of the five studies in children [20–22,24] had adjusted for presence of a TB contact in the household, and the association was found to be stronger (RR 2.79, 95% CI 2.02–3.84); meta-regression analysis indicated that the difference between presence and absence of TB contact had statistical significance (*p =* 0.045) (S2 Table). Meta-regression analysis did not show any other significant effect size modification by the specific study characteristics considered, possibly because of a relatively small number of studies.

### Second-Hand Smoke Exposure and Active TB Disease

Twelve of the 18 studies [18,25–35] discussed the risk of active TB with exposure to SHS (Table 3). The pooled estimate for all 12 studies showed a nearly 2-fold increased risk of TB disease from SHS exposure (pooled RR 1.96, 95% CI 1.37–2.80). The majority of the studies were rated as good quality (10/12, with RR 2.18, 95% CI 1.43–3.32), were case-control studies (10/12, RR 2.32, 95% CI 1.45–3.70), discussed pulmonary TB only (8/12, RR 1.66, 95% CI 1.07–2.57), and used microbiological diagnosis (8/12, RR 1.74, 95% CI 1.15–2.64). Half of the studies [27–29,33–35] were from the South-East Asia Region (RR 2.61, 95% CI 1.26–5.40). Further subgroup analysis revealed that case-control studies with community-based controls showed a notably a stronger association of SHS exposure with active TB (RR 4.90, 95% CI 2.15–11.17, *p* for heterogeneity = 0.387, *I* ^2^ = 0.0%) compared to studies with hospital-based controls (RR 1.96, 95% CI 1.16–3.31, *p* for heterogeneity = 0.017, *I* ^2^ = 66.8%) or other controls (RR 2.01, 95% CI 0.56–7.20, *p* for heterogeneity = 0.004, *I* ^2^ = 82.3%). The stratified analysis for the presence of a TB patient in the household (7/12 studies [26,27,29,30,33–35]) showed a significant association of SHS with active TB disease (RR 3.07, 95% CI 1.82–5.16). After adjusting for TB contacts in the household (3/7 studies [27,29,33]), risk attenuated but remained statistically significant (RR 1.87, 95% CI 1.30–2.69, *p* for heterogeneity = 0.693, *I* ^2^ = 0.0%). The association remained positive and statistically significant even after adjustments for both age and SES (RR 2.13, 95% CI 1.18–3.83, *p* for heterogeneity < 0.001, *I* ^2^ = 71.4%) and for BMF (RR 2.03, 95% CI 1.13–3.63, *p* for heterogeneity = 0.004, *I* ^2^ = 73.9%), although significant heterogeneities were observed. Of study characteristics assessed in meta-regression, studies with a pediatric population (*p =* 0.028) and presence of a TB patient in the household (*p =* 0.029) were statistically significant (Table 3). Meta-regression also showed marginal significance (*p =* 0.105) when a microbiological mode of diagnosis was used for TB outcome compared with other modes of diagnosis.

**Table 3:** Quality assessment and subgroup analysis: second-hand smoke exposure and active TB disease.

Measure or Outcome	Study Characteristic (Number of Studies)	Summary Estimate	95% CI	*I* ^*2*^ (95% CI)	Meta-Regression	
					Coefficient (95% CI)	*p*-Value
**Population**	All studies (12) [18, 25–35]	1.96	1.37–2.80	68.1 (45.4%–81.3%)	(..)	(..)
	Children (7) [18,26,29,31,33–35]	3.41	1.81–6.45	72.1 (39.6%–87.1%)	0.89 (0.11–1.67)	0.028
	Adults (6) [25,27,28,30–32]	1.32	1.04–1.68	42.3 (0.0%–74.5%)	Ref	
**Sex**	Both (10) [18,25–29,31,33–35]	1.97	1.25–3.09	71.3% (49.6%–83.6%)	Ref	
	Male (1) [32]	2.53	0.60–10.62	(..)	−0.38 (−2.09, 1.33)	0.636
	Female (1) [30]	2.70	0.77–9.48	(..)	−0.56 (−2.34, 1.22)	0.506
**Quality of study**	Good (10) [25–31,33–35]	2.18	1.43–3.32	70.9% (48.9%–83.5%)	0.58 (−0.64, 1.81)	0.321
	Poor to moderate (2) [18,32]	1.22	0.79–1.90	0.0%	Ref	
**WHO region**	EUR (3) [25,26,32]	2.49	1.01–6.14	76.5% (23.1%–92.9%)	0.73 (−1.26, 2.72)	0.438
	SEAR (6) [27–29,33–35]	2.61	1.26–5.40	73.7% (39.8%–88.5%)	0.77 (−1.11, 2.65)	0.386
	WPR (2) [30,31]	1.34	0.80–2.24	51.7% (0.0%–82.3%)	0.19 (−1.72, 2.09)	0.834
	AMR (1) [18]	1.20	0.63–2.30	(..)	Ref	
**Outcome**	Pulmonary TB only (8) [18,25–29,31,32]	1.66	1.07–2.57	62.2% (27.2%–80.3%)	0.57 (−0.39, 1.53)	0.222
	Pulmonary/extra-pulmonary TB (4) [30,33–35]	2.92	1.46–5.86	80.6% (48.9%–92.6%)	Ref	
**Multivariate adjusted analysis**	Yes (10) [25–31,33–35]	2.18	1.43–3.32	70.9% (48.9%–83.5%)	0.58 (−0.64, 1.81)	0.321
	No (2) [18,32]	1.22	0.79–1.90	0.0%	Ref	
**Adjustment for age**	Yes (10) [25–31,33–35]	2.18	1.43–3.32	70.9% (48.9%–83.5%)	0.58 (−0.64, 1.81)	0.321
	No (2) [18,32]	1.22	0.79–1.90	0.0%	Ref	
**Adjustment for alcohol intake (adults)**	Yes (2) [30,31]	1.26	0.94–1.70	49.1% (0.0%–83.2%)	−0.49 (−1.44, 0.46)	0.286
	No (4) [25,27,28,32]	1.44	0.96–2.17	49.7% (0.0%–83.4%)	Ref	
**Adjustment for SES**	Yes (8) [25,26,28–31,33,34]	1.91	1.24–2.93	68.6% (41.1%–83.2%)	−0.11 (−1.15, 0.93)	0.827
	No (4) [18,27,32,35]	2.17	0.99–4.74	75.1% (31.1%–91.0%)	Ref	
**Adjustment for age and SES**	Yes (7) [25–29,31,34]	2.13	1.18–3.83	71.4% (45.6%–85.0%)	0.12 (−0.84, 1.08)	0.786
	No (5) [18,30,32,33]	1.74	1.13–2.68	66.1% (11.5%–87.0%)	Ref	
**Adjustment for cooking/BMF**	Yes (7) [18,28,29,32–35]	2.03	1.13–3.63	73.9% (44.2%–87.8%)	0.03 (−0.91, 0.97)	0.948
	No (5) [25–27,30,31]	1.93	1.17–3.17	66.3% (28.6%–84.1%)	Ref	
**Adjustment for BCG vaccination**	Yes (2) [26,27]	3.81	2.09–6.96	42.6%	0.71 (−0.57, 2.00)	0.252
	No (10) [18,25,28–35]	1.76	1.22–2.53	65.2% (37.3%–80.7%)	Ref	
**Type of study**	Cohort (2) [30,31]	1.34	0.80–2.24	51.7% (0.0%–82.3%)	−0.49 (−1.44, 0.46)	0.286
	Case-control (10) [18,25–29,32–35]	2.32	1.45–3.70	70.3% (43.2%–84.5%)	Ref	
**Type of control among case-control studies**	Community-based (2) [29,34]	4.90	2.15–11.17	0.0%	0.78 (−1.29, 2.85)	0.404
	Hospital-based (5) [18,27,32,33,35]	1.96	1.16–3.31	66.8% (13.8%–87.2%)	−0.01 (−1.3, 2.9)	0.997
	Close contacts of cases (3) [25,26,28]	2.01	0.56–7.20	82.3% (45.5%–94.2%)	Ref	
**TB contact in the household**	Yes (7) [26,27,29,30,33–35]	3.07	1.82–5.16	71.9% (39.0%–87.0%)	0.86 (0.10, 1.62)	0.029
	No (5) [18,25,28,31,32]	1.18	0.89–1.57	41.8% (0.0%–74.3%)	Ref	
**Adjustment for TB contact in the household**	Yes (3) [27,29,33]	1.87	1.30–2.69	0.0% (0.0%–89.6%)	−0.64 (−2.11, 0.82)	0.31
	No (4) [26,30,34,35]	4.33	1.64–11.46	84.5% (61.3%–93.8%)	Ref	
**Mode of diagnosis**	Microbiological (8) [18,25–30,32]	1.74	1.15–2.64	58.1% (8.3%–80.9%)	−1.04 (−2.34, 0.25)	0.105
	Radiographic findings/TST (1) [31]	1.38	0.63–3.04	58.1% (0.0%–86.1%)	−0.80 (−1.90, 0.29)	0.136
	Other (3) [33–35]	4.29	1.35–13.65	83.3% (49.5%–94.5%)	Ref	

AFR, African Region; AMR, Region of the Americas; BCG, Bacillus Calmette–Guérin EUR, European Region; SEAR, South-East Asia Region; TST, tuberculin skin test. “(..)” indicates that the nominated reference group was not available to compute meta-regression statistics.

In subgroup analysis, children showed a more than 3-fold increased risk of SHS-associated active TB (RR 3.41, 95% CI 1.81–6.45), which was also higher than the risk in adults (RR 1.32, 95% CI 1.04–1.68) (S3 Table). In children exposed to SHS, risk of pulmonary TB was three times as high (RR 2.96, 95% CI 1.11–7.85) as in those not exposed to SHS. Risk of pulmonary or extra-pulmonary TB disease was even greater (RR 4.29, 95% CI 1.35–13.65) and was also higher than in adults exposed to SHS (RR 1.49, 95% CI 1.01–2.19). We found strong associations between SHS exposure and active TB, particularly for children, in the stratified analysis by outcome definition, study design, control population (for case-control studies), multivariate adjustment, and mode of diagnosis (S3 Table). Stratification by most of these study-specific variables did not fully explain the variability but partially accounted for the heterogeneity. The association with SHS exposure was stronger for pulmonary or extra-pulmonary TB disease than for pulmonary TB alone in both men and women. However, effect estimates for pulmonary TB were not statistically different from those for any TB disease (*p =* 0.222) (see Table 3). Among studies that reported pulmonary/extra-pulmonary TB, pulmonary TB represented 86% of total active TB cases (72% among children and 97% among adults). The heterogeneity among these studies was largely explained by the age of the populations. Four of seven studies among children [26,33–35] showed strong associations between SHS exposure and active TB, although there was significant heterogeneity among studies (*I* ^2^ = 72.1%) (Fig 2B). Among adults, five out of six studies [25,27,28,30,32] used microbiological testing as the mode of diagnosis (RR 1.47, 95% CI 1.11–1.94) and showed low heterogeneity (*I* ^2^ = 33.1%). Meta-regression for adult and child subgroup analyses showed no significant effect size modification by most study-specific variables, except for adjusted estimates in children living with a TB contact in the household (*p =* 0.033).

### Factors Showing a Dose–Response Relation with Risk of Active TB Disease

Among those exposed to SHS who developed active TB disease, several socio-demographic and household factors showed a dose–response relation. For children, these factors included age, frequency and closeness of contact with a TB patient in the household, household crowdedness, and relationship with the smoker. For adults, these factors were age, contact with a TB patient in the household, number of smokers in the household, and number of cigarettes smoked by household members.

**Fig 3:**
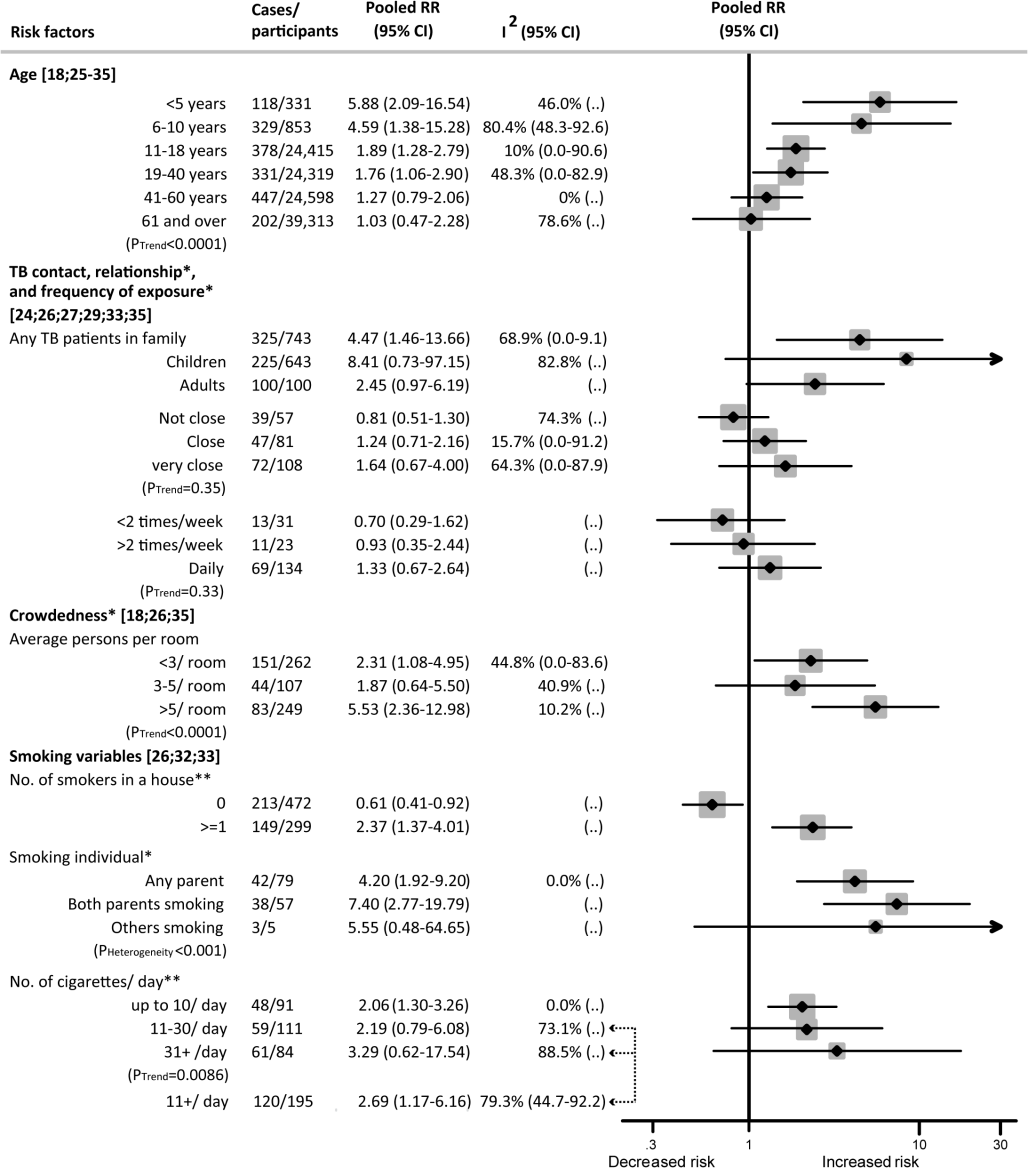
Dose–response relationship between second-hand smoke exposure and active TB disease in children and adults. Weights are from random-effects analysis. “(..)” indicates that not enough studies were available to compute *I* ^2^ or confidence intervals around *I* ^2^. *Children only. **Adults only.

While each age group showed an increased risk of TB disease with exposure to SHS, children aged 0–5 y showed the highest relative risk (RR 5.88, 95% CI 2.09–16.54) (Fig 3). Relative risk decreased progressively with age (*p* trend < 0.001). Within the adult population, those aged 19–40 y were at a higher risk than older adults (RR 1.76, 95% CI 1.06–2.90).

Those exposed to SHS and in contact with a TB patient in the household had a greater than four times higher risk of developing active TB (RR 4.47, 95% CI 1.46–13.66) than those unexposed to SHS and without a TB patient in the household. When stratified by sub-populations, SHS exposure was associated with an 8-fold increase in the risk of active TB disease in children (RR 8.41, 95% CI 0.73–97.15) and a 2.5-fold increase in adults (RR 2.45, 95% CI 0.97–6.19), with marginal significance (Fig 3). For children exposed to SHS versus those not exposed, the relative risk of active TB increased progressively, but not significantly, with closeness of contact to a TB patient (RR 1.64, 95% CI 0.67–4.00) and daily exposure to a TB contact (RR 1.33, 95% CI 0.67–2.64) compared to those with less closeness or less frequent exposure. A clear dose–response was found when stratified by both crowdedness and exposure intensity to SHS. SHS exposure in children living in households with more than five people per room showed a significantly higher association with active TB (RR 5.53, 95% CI 2.36–12.98, *p* trend < 0.001) than SHS exposure in those living in households with fewer people per room. Similar risk was observed for children with one parent (RR 4.20, 95% CI 1.92–9.20) or both parents (RR 7.40, 95% CI 2.77–19.79) smoking (*p* for heterogeneity < 0.001).

Among non-smoking adults, a linear, increasing dose–response was observed for the frequency of smokers in their household and the smoking quantity of those smokers (*p* trend = 0.0086). Any number of smokers in the household was strongly associated with an increased risk of TB (RR 2.37, 95% CI 1.37–4.01), while having no smokers in the household was not (RR 0.61, 95% CI 0.41–0.92). SHS exposure to those smoking ten cigarettes or more per day was significantly associated with a higher risk of TB (RR 2.69; 95% CI 1.17–6.16) (Fig 3).

### Publication Bias

**Fig 4:**
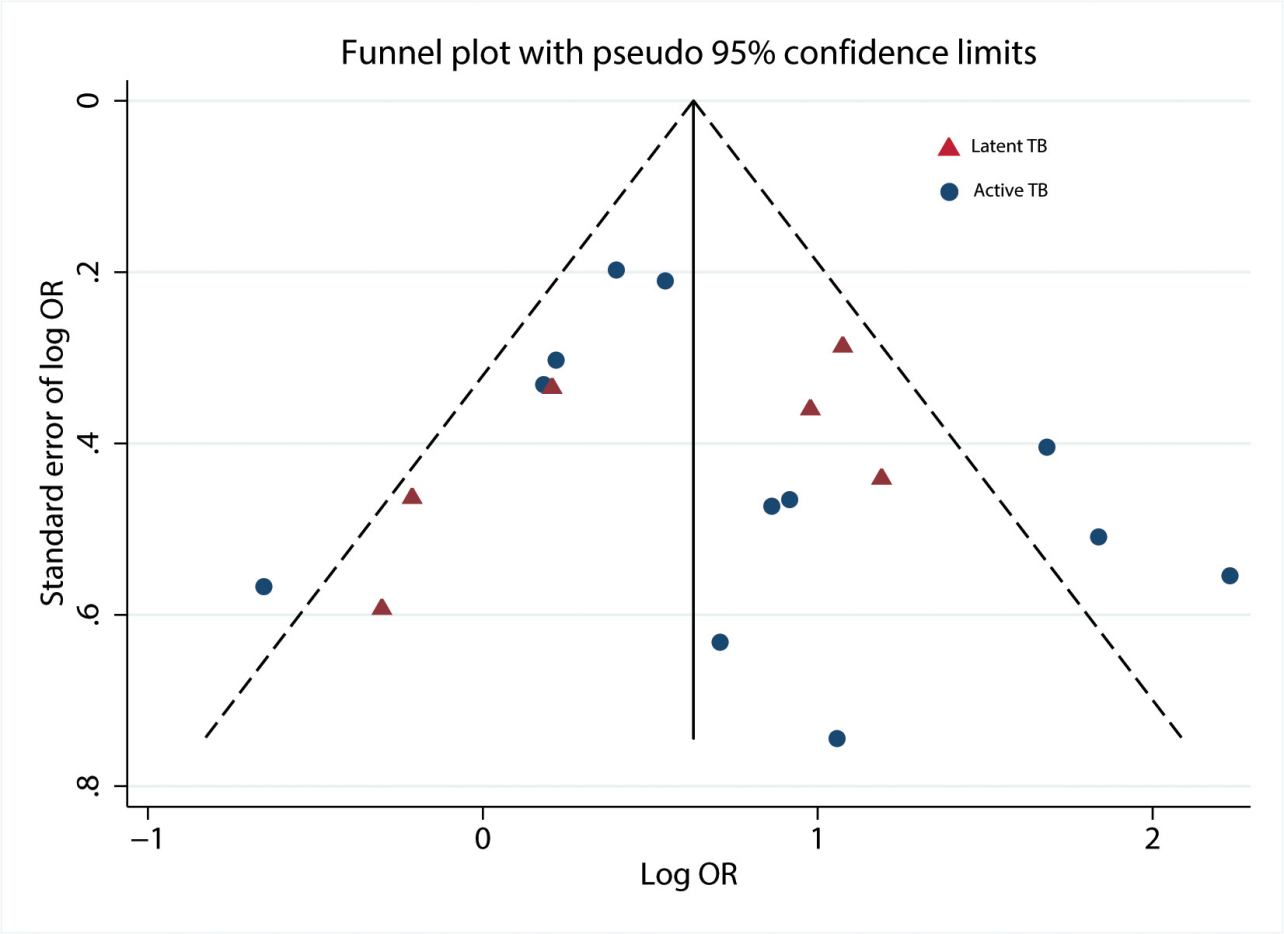
Funnel plot with pseudo 95% confidence limits. OR, odds ratio.

When we plotted the natural logarithms of the effect estimates against their standard errors (Fig 4), we detected very little asymmetry of effect estimates among small studies on TB disease [26,28,34,35]. We also noted that one data point for a large study on LTBI [22] fell outside the boundary of the funnel plot and Galbraith plot. Exclusion from the analysis decreased heterogeneity scores overall and among children. No evidence for substantial publication bias was found by the Begg’s test (*p* = 0.37) or Egger’s test (*p* = 0.79).

## Discussion

In this meta-analysis, SHS exposure appeared to be associated with LTBI and active TB after adjustment for a limited number of factors, although one caveat is that the possibility of confounding could not be completely ruled out, especially in the stratified analyses for SES and the study quality. Among the studies with adjusted measures, we found that SHS exposure was associated with an increased relative risk of TB and LTBI infection after controlling for age, BMF use, contact with a TB patient, SES, and study quality; the association of SHS exposure with LTBI was significant after adjustments for the above factors, except for SES and study quality. Much of the evidence on the biological mechanism linking SHS exposure to TB development concerns the effects of mainstream cigarette smoke, i.e., residual smoke exhaled upon cigarette smoking [10]. However, sidestream smoke that is released from a burning cigarette can also adversely affect the pulmonary mechanical barriers and immune system to influence TB disease development [10,11]. Prolonged exposure to cigarette smoke damages the first line of defense against Mtb by inducing growth of abnormal cilia on the pulmonary lining. These cilia may impair the mucociliary clearance process that is responsible for trapping contaminants such as Mtb in the respiratory tract [10]. The pulmonary immune system in smokers is also weakened because of reduced T cell proliferation and suboptimal functioning of alveolar macrophages [10], which can also lead to increased susceptibility to Mtb infection and, subsequently, progression to TB disease.

Regional variations were found in the risk of LTBI and active TB. The highest risk was observed in South-East Asia and sub-Saharan Africa. This is not surprising considering that certain countries comprising these regions and represented in this analysis, specifically India, South Africa, and Thailand, are among those with the highest burden of TB disease [3,4]. It is interesting to note that a high risk of active TB due to SHS exposure was also found in the studies conducted in Spain. Spain also has a high prevalence of SHS exposure in both children and adults compared to the US, indicating that even in the setting of a developed economy, exposure to SHS may be related to the risk of developing active TB [36].

The high risk for children under 5 y, and the increase in risk for both children and adults, with number of smokers in the household and household crowdedness shows that when exposed to SHS, household/environmental factors may increase the risk of TB. Mainstream and sidestream smoke in households is the major source of SHS exposure for children, and living in a crowded house with adult smokers, especially parents, prolongs children’s exposure. The increased risk of TB disease presented in this meta-analysis supports the association between SHS exposure and active TB disease [37].

### Strengths and Limitations

To our knowledge, this is the first systematic review and meta-analysis of the epidemiological evidence on the association between SHS exposure and latent/active TB in children and adults. We assessed sensitivity to study design, methods, and quality indicators using meta-regression and subgroup analysis, and we searched multiple databases without language limitation for our systematic review. The major limitation of this analysis is the high heterogeneity in outcomes among the studies investigating pediatric cases of TB disease. Sputum is difficult to obtain in children, and TB disease is largely paucibacillary, which makes microbiological diagnosis in children a challenge. The studies in this meta-analysis are highly variable in terms of the diagnostic tests (microbiological, clinical, and/or radiological) used to diagnose TB in children. There is a possibility of confounding in our meta-analysis since all the studies we included were observational. The major potential confounding factors included age, SES, BMF use, and contact with a TB patient in the household. Newborns, young adults, and socioeconomically disadvantaged individuals, including those frequently using BMF, share a larger burden of TB disease [38,39] and a higher exposure to SHS [36,40]. To control for the effect of these potential confounders, we conducted sensitivity analyses of studies that adjusted for these variables. The association between SHS exposure and TB disease remained after adjustment for a limited number of factors (age, BMF use, and TB contact in the household). However, the effect size attenuated after adjustment for SES; this highlights the confounding role of SES, and is also consistent with the existing evidence showing a higher burden of TB in low- and middle-income countries [35]. In addition, these studies do not capture the variability in household air pollution, which is affected by the type of BMF used, kitchen ventilation, and indoor versus outdoor tobacco smoking—factors that affect exposure to SHS and are linked to TB disease [38]. It is challenging to decipher the exact nature of the association between SHS exposure and TB given the relatively few studies that have been published on this topic; however, this only indicates a greater need to further explore the impact of SHS exposure, whether as an independent risk factor or as a factor that works in conjunction with other risk factors, such as BMF exposure and contact with a TB patient, to exacerbate their impact on the risk of TB disease.

### Implications

Despite low comparability between studies, this meta-analysis lends to future hypotheses exploring the role of SHS exposure in TB disease, particularly in children and in low- and middle-income countries that have a greater prevalence of SHS exposure. Future studies should aim to use nationally representative datasets to rigorously explore the effect of BMF use on SHS exposure, diverse (and perhaps more reliable) measures of SHS exposure such as hair cotinine levels [41], and pediatric populations with standardized TB diagnostic measures. Exposure to SHS and its impact on TB should be explored further, given that TB deaths are projected to increase from 2010 to 2050 by as much as 167%, as found in a mathematical modelling analysis [42]. Further examination of this association and the potential for subsequent efforts to reduce SHS exposure along with TB control efforts will have important implications for reducing TB incidence and deaths. Probing potential links between SHS exposure and TB may have important implications for TB and tobacco control programs, especially for children in settings with high SHS exposure and TB burden [42,43].

## Supporting Information

S1 Protocol Study protocol for systematic review and meta-analysis to determine the relation between exposure to second-hand smoke and tuberculosis. (DOC)

S1 Table Quality assessment using Newcastle–Ottawa scale. (PDF)

S2 Table Quality assessment and subgroup analysis: second-hand smoke exposure and latent TB infection by population. (PDF)

S3 Table Quality assessment and subgroup analysis: second-hand smoke exposure and active TB by population. (PDF)

S1 Text PRIMA checklist for the meta-analysis. (DOC)

S2 Text Newcastle–Ottawa quality assessment scale for cohort, case-control, and cross-sectional (adapted) studies. (DOC)

## Abbreviations:

BMF: biomass fuel
CVD: cardiovascular disease
ETS: environmental tobacco smoke
LTBI: latent tuberculosis infection
Mtb: Mycobacterium tuberculosis
RR: relative risk
SES: socioeconomic status
SHS: second-hand smoke
TB: tuberculosis

## References

[1] Lozano R, Laghavi M, Foreman K, Lim S, Shibuya K, et al Global and regional mortality from 235 causes of death for 20 age groups in 1990 and 2010: a systematic analysis for the Global Burden of Disease Study 2010. Lancet. 2012;380:2095–2128. 10.1016/S0140-6736(12)61728-0 23245604 PMC10790329

[2] World Health Organization Health statistics and information systems: estimates for 2000–2012—cause specific mortality Geneva: World Health Organization; 2015 http://www.who.int/healthinfo/global_burden_disease/estimates/en/index1.html. Accessed May 4, 2015.

[3] World Health Organization. Global tuberculosis report 2014 Geneva: World Health Organization; 2014 http://www.who.int/tb/publications/global_report/en/. Accessed May 4, 2015.

[4] Dodd PJ, Gardiner E, Coghlan R, Seddon JA. Burden of childhood tuberculosis in 22 high-burden countries: a mathematical modelling study. Lancet Glob Health. 2014;2:e453–e459. 10.1016/S2214-109X(14)70245-1 25103518

[5] World Health Organization. Roadmap for childhood tuberculosis: towards zero deaths Geneva: World Health Organization; 2013 http://apps.who.int/iris/bitstream/10665/89506/1/9789241506137_eng.pdf. Accessed November 10, 2014.

[6] Patra J, Jha P, Rehm J, Suraweera W. Tobacco smoking, alcohol drinking, diabetes, low body mass index and the risk of self-reported symptoms of active tuberculosis: individual participant data (IPD) meta-analyses of 72,684 individuals in 14 high tuberculosis burden countries. PLoS ONE. 2014;9:e96433 10.1371/journal.pone.0096433 24789311 PMC4008623

[7] Lin HH, Ezzati M, Murray M. Tobacco smoke, indoor air pollution and tuberculosis: a systematic review and meta-analysis. PLoS Med. 2007;4:e20 17227135 10.1371/journal.pmed.0040020 PMC1769410

[8] Hodge S, Hodge G, Ahem J, Jersmann H, Holmes M, Reynolds PN. Smoking alters alveolar macrophage recognition and phagocytic ability implications in chronic obstructive pulmonary disease. Am J Respir Cell Mol Biol. 2007;37:748–755. 17630319 10.1165/rcmb.2007-0025OC

[9] Shang S, Ordway O, Henao-Tamayo M, Bai X, Oberley-Deegan R, Shanley C, et al Cigarette smoke increases susceptibility to tuberculosis—evidence from in vivo and in vitro models. J Infect Dis. 2011;203:1240–1248. 10.1093/infdis/jir009 21357942

[10] Sopori M. Effects of cigarette smoke on the immune system. Nat Rev Immunol. 2002;2:372–377. 12033743 10.1038/nri803

[11] Stampfli MR, Anderson GP. How cigarette smoke skews immune responses to promote infection, lung disease and cancer. Nat Rev Immunol. 2009;9:377–384. 10.1038/nri2530 19330016

[12] Wells GA, Shea B, O’Connell D, Peterson J, Welch V, Losos M., et al The Newcastle-Ottawa Scale (NOS) for assessing the quality of nonrandomized studies in meta-analysis Ottawa Hospital Research Institute; 2011 http://www.ohri.ca/programs/clinical_epidemiology/oxford.asp. Accessed February 2, 2015.

[13] Moher D, Liberati A, Tetzlaff J, Altman DG. Preferred reporting items for systematic reviews and meta-analyses: the PRISMA statement. Ann Intern Med. 2009;151:264–269. 19622511 10.7326/0003-4819-151-4-200908180-00135

[14] DerSimonian R, Laird N. Meta-analysis in clinical trials. Control Clin Trials. 1986;7:177–188. 3802833 10.1016/0197-2456(86)90046-2

[15] Higgins JPT, Thompson SG. Quantifying heterogeneity in a meta-analysis. Stat Med. 2002;21:1539–1558. 12111919 10.1002/sim.1186

[16] Galbraith RF. A note on graphical presentation of estimated odds ratios from several clinical trials. Stat Med. 1988;7:889–894. 3413368 10.1002/sim.4780070807

[17] Egger M, Smith GD, Schneider M, Minder C. Bias in meta-analysis detected by a simple, graphical test. BMJ. 1997;315:629–634. 9310563 10.1136/bmj.315.7109.629 PMC2127453

[18] García-Sancho C, Fernandez-Plata R, Martinez-Briseno D, Torre-Bouscoulet L, Gochicoa-Rangel L, Franco-Marina F, et al Exposure to wood smoke and tuberculosis in children. Neumol Cir Torax. 2013;72:281–286.

[19] Babayigit-Hocaoglu A, Olmez-Erge D, Anal O, Makay B, Uzuner N, Karaman O. Characteristics of children with positive tuberculin skin test. Tuberk Toraks. 2011;59:158–163. 21740391

[20] den Boon S, Verver S, Marais BJ, Enarson DA, Lombard CJ, Bateman ED, et al Association between passive smoking and infection with Mycobacterium tuberculosis in children. Pediatrics. 2007;119:734–739. 17403844 10.1542/peds.2006-1796

[21] Du Preez K., Mandalakas AM, Kirchner HL, Grewal HM, Schaaf HS, van Wyk SS, et al Environmental tobacco smoke exposure increases Mycobacterium tuberculosis infection risk in children. Int J Tuberc Lung Dis. 2011;15:1490–1496. 10.5588/ijtld.10.0759 22008762

[22] Lindsay RP, Shin SS, Garfein RS, Rusch MLA, Novotny TE. The association between active and passive smoking and latent tuberculosis infection in adults and children in the United States: results from NHANES. PLoS ONE. 2014;9:e93137 10.1371/journal.pone.0093137 24664240 PMC3963991

[23] Shin SS, Laniado-Laborin R, Moreno PG, Novotny TE, Strathdee SA, Garfein RS. Dose-response association between salivary cotinine levels and Mycobacterium tuberculosis infection. Int J Tuberc Lung Dis. 2013;17:1452–1458. 10.5588/ijtld.13.0311 24125450 PMC3801225

[24] Singh M, Mynak ML, Kumar L, Mathew JL, Jindal SK. Prevalence and risk factors for transmission of infection among children in household contact with adults having pulmonary tuberculosis. Arch Dis Child. 2005;90:624–628. 15908630 10.1136/adc.2003.044255 PMC1720464

[25] Alcaide J, Altet MN, Plans P, Parron I, Folguera L, Salto E, et al Cigarette smoking as a risk factor for tuberculosis in young adults: a case-control study. Tuber Lung Dis. 1996;77:112–126. 8762844 10.1016/s0962-8479(96)90024-6

[26] Altet MN, Alcaide J, Plans P, Taberner JL, Salto E, Folguera L, et al Passive smoking and risk of pulmonary tuberculosis in children immediately following infection. A case-control study. Tuber Lung Dis. 1996;77:537–544. 9039447 10.1016/s0962-8479(96)90052-0

[27] Ariyothai N, Podhipak A, Akarasewi P, Tornee S, Smithtikarn S, Thongprathum P. Cigarette smoking and its relation to pulmonary tuberculosis in adults. Southeast Asian J Trop Med Public Health. 2004;35:219–227. 15272772

[28] Gupta D, Vinay N, Agarwal R, Agarwal AN. Socio-demographic profile of patients with sarcoidosis vis-a-vis tuberculosis. Sarcoidosis Vasc Diffuse Lung Dis. 2013;30:186–193. 24284291

[29] Jubulis J, Kinikar A, Ithape M, Khandave M, Dixit S, Hotalkar S, et al Modifiable risk factors associated with tuberculosis disease in children in Pune, India. Int J Tuberc Lung Dis. 2014;18:198–204. 10.5588/ijtld.13.0314 24429313 PMC4487622

[30] Leung CC, Lam TH, Ho KS, Yew WW, Tam CM, Chan WM, et al Passive smoking and tuberculosis. Arch Intern Med. 2010;170:287–292. 10.1001/archinternmed.2009.506 20142576

[31] Lin HH, Chiang YT, Chuang JH, Yang SL, Chang HY, Ezzati M, et al Exposure to secondhand smoke and risk of tuberculosis: prospective cohort study. PLoS ONE. 2013;8:e77333 10.1371/journal.pone.0077333 24204811 PMC3808396

[32] Oztürk AB, Kilicaslan Z, Issever H. Effect of smoking and indoor air pollution on the risk of tuberculosis: smoking, indoor air pollution and tuberculosis. Tuberk Toraks. 2014;62:1–6. 24814071 10.5578/tt.7013

[33] Patra S, Sharma S, Behera D. Passive smoking, indoor air pollution and childhood tuberculosis: a case control study. Indian J Tuberc. 2012;59:151–155. 23362712

[34] Ramachandran R, Indu PS, Anish TS, Nair S, Lawrence T, Rajasi RS. Determinants of childhood tuberculosis—a case control study among children registered under revised national tuberculosis control programme in a district of South India. Indian J Tuberc. 2011;58:204–207. 22533171

[35] Tipayamongkholgul M, Podhipak A, Chearskul S, Sunakorn P. Factors associated with the development of tuberculosis in BCG immunized children. Southeast Asian J Trop Med Public Health. 2005;36:145–150. 15906658

[36] Oberg M, Jaakkola M, Woodward A, Peruga A, Pruss-Ustun A. Worldwide burden of disease from exposure to second-hand smoke: a retrospective analysis of data from 192 countries. Lancet 2011;377:139–146. 10.1016/S0140-6736(10)61388-8 21112082

[37] Seddon JA, Shingadia D. Epidemiology and disease burden of tuberculosis in children: a global perspective. Infect Drug Resist. 2014;18:153–165. 10.2147/IDR.S45090 PMC4069045 24971023

[38] Fullerton DG, Bruce N, Gordon SB. Indoor air pollution from biomass fuel smoke is a major health concern in the developing world. Trans R Soc Trop Med Hyg. 2008;102:843–851. 10.1016/j.trstmh.2008.05.028 18639310 PMC2568866

[39] Gordon SB, Bruce NG, Grigg J, Hibberd PL, Kurmi OP, Lam KB, et al Respiratory risks from household air pollution in low and middle income countries. Lancet Respir Med. 2014;2:823–860. 10.1016/S2213-2600(14)70168-7 25193349 PMC5068561

[40] Pisinger C, Helmich LH, Andreasen AH, Jogensen T, Glumer C. Social disparities in children’s exposure to second hand smoke at home: a repeated cross-sectional survey. Environ Health. 2012;11:65 10.1186/1476-069X-11-65 22984822 PMC3544183

[41] Al-Delaimy WK, Crane J, Woodward A. Is the hair nicotine level a more accurate biomarker of environmental tobacco smoke exposure than urine cotinine? J Epidemiol Community Health. 2002;56:66–71. 11801622 10.1136/jech.56.1.66 PMC1732006

[42] Basu S, Stuckler D, Bitton A, Glantz SA. Projected effects of tobacco smoking on worldwide tuberculosis control: mathematical modelling analysis. BMJ. 2011;343:d5506 10.1136/bmj.d5506 21972295 PMC3186817

[43] World Health Organization A WHO/The Union monograph on TB and tobacco control: joining efforts to control two related global epidemics World Health Organization; 2007 http://www.who.int/tobacco/resources/publications/tb_tob_control_monograph/en. Accessed Feb 19, 2015.

