# Supplementary material for: Exposure to Second-Hand Smoke and the Risk of Tuberculosis in Children and Adults: A Systematic Review and Meta-Analysis of 18 Observational Studies
Source: PLoS Med. 2015 Jun 2;12(6):e1001835. doi: 10.1371/journal.pmed.1001835 (PMC4452762; doi:10.1371/journal.pmed.1001835)
Supplement: S1 Protocol — (DOC) [file pmed.1001835.s001.doc]

**S1 Protocol** Study protocol for systematic review and meta-analysis to determine the relation between exposure to second-hand smoke (SHS) and tuberculosis (TB)*

** Amendments were suggested during peer-review process and were included in the subsection below where necessary in italics.*

Patra et al. **“Exposure to second-hand smoke and the risk of tuberculosis in children and adults: systematic review and a meta-analysis of 18 observational studies”**

**Objective**

In this systematic review and meta-analysis, we aim to investigate the role of SHS exposure as a risk factor for TB and estimated SHS–attributable TB among children and adults.

**Inclusion criteria**

*Study type*

- All study types will be eligible to enter meta-analysis.
- We will include all studies which were published as original report and present information on the relation between SHS and TB (latent TB and active TB).

*Participants*

- Children and adults of all age groups will be included.
- We will perform sub-group analyses (e.g. different age groups, geography, etc.).

*Definition of exposition*

- All studies which reported information on SHS will be included as well as studies which reported odds ratios (OR) and 95% confidence intervals (95% CI) (or data with which to calculate them) for risk of TB.
- Within SHS we accept frequency of exposure to SHS for dose-response relationship.

*Outcome variable*

- All studies which reported risk of latent and active TB will be included.
- TB type such as pulmonary and extra-pulmonary TB will be stratified*.*
- Diagnosis of TB will also be used as an outcome variable.

Outcome measures

- The OR will either be extracted from the published article or calculated by the authors.
- If the OR is not directly reported or cannot be readily extracted from the published data, the reviewers will contact the corresponding authors for additional information (e.g., data provided in 2x2 contingency tables).

*Publication type*

- Full published papers will be eligible (language restrictions were applied to English,

Spanish and Chinese (mandarin)).

*Amendment(s): Full published papers excluding case reports, review articles, and editorials will be eligible. No language restrictions were applied.*

**Search Methods**

We will search the following electronic databases:

- MedLine (via PubMed)
- EMBASE
- Scopus
- Web of Science
- Google Scholar

There will be no restriction on year and language of publication. In these databases, we will search according to the thesaurus of the NCBI MESH browser the following terms and combinations of keywords in full text:

The following keywords will be employed:

1. ‘‘tuberculosis’’

2. ‘‘second-hand smoking’’

3. ‘‘environmental exposure’’

4. ‘‘passive smoking’’

5. ‘‘tobacco pollution’’

6. ‘‘cohort studies’’ OR ‘‘case -control studies’’ OR ‘‘cross -sectional studies’’

OR ‘‘epidemiologic studies’’ OR ‘‘prospective studies’’ OR ‘‘ratio’’ OR

“risk”

7. ‘‘(1) AND (2) AND (6)” OR ‘‘(1) AND (3) AND (6)” OR ‘‘(1) AND (4) AND (6)” OR ‘‘(1) AND (5) AND (6)’’

Additionally, bibliographies of identified publications and published reviews will be hand searched for potentially relevant articles. Authors will be contacted if data, methods and/or parameter definitions provided from the respective studies are unclear.

**Reviews**

All references cited in the identified reviews will be manually searched for potentially relevant studies.

**Data collection**

Two reviewers (JP, MB) will independently scrutinize the list of titles, and if available the abstracts, to determine potential usefulness of the article. Final selection will be based on the full text of potentially relevant articles by the two reviewers independently. In cases of disagreement, both authors will review the materials together until a consensus is reached. Study quality will be measured using the Newcastle–Ottawa scale (Wells et al. 2011).

The following study characteristics will be extracted: publication year, country, study design, average year, age at outcome, cohort size, assessment of TB, assessment of SHS, main result.

From all eligible studies, relevant data will be abstracted in duplicate, using a standardized data extraction sheet. An independent reviewer will confirm all data entries and will check at least twice for completeness and accuracy.

*Amendment(s): A modified version of Newcastle-Ottawa scale for cross-sectional study was used to rate quality indicators.*

**Meta-analysis & Meta-regression**

*Dichotomous comparisons*

- Data on numbers of subjects with and without TB through SHS and corresponding crude odds ratios and 95% confidence intervals will be calculated.
- Random-effects models to estimate the pooled odds ratios for risk of TB due to exposure to SHS will be constructed across all studies.

*Amendment(s): The value of the I^2^ statistic was used to select the appropriate pooling method: fixed-effects models were used for I^2^<50% and random-effects models for I^2^>=50%.*

*Dichotomous comparisons*

- Dose-response relationship (Berlin et al 1993) will be measured through exposure intensity of SHS (age, TB contact, relationship, frequency of exposure, crowdedness, and smokers and amount of smoking in family).

*Assessment of heterogeneity*

- Impact of heterogeneity will be assessed by calculating the I^2^ according to Higgins et al (Higgins JP et al. 2003).

*Amendment(s): Confidence intervals around the I^2^ were also provided.*

*Influence analysis*

- Robustness of the pooled estimates will be checked by influence analyses. Each of the studies will be individually omitted from the data set, followed in each case by recalculation of the pooled estimate of the remaining studies.

*Subgroup/Sensitivity analyses*

- To identify potential sources of heterogeneity and sources of bias, studies will be stratified by study design and assessment of birth weight data to assess potential recall bias.
- Further stratifications will be made by geographic origin, TB outcome, population type (children or adults), adjustment for confounders, mode of diagnosis, type of study (cohort or case control), type of control among case control studies, and exposure intensity of SHS (crowdedness, smokers in family, TB contact).

*Amendment(s): Meta-regression was used to evaluate whether effect size estimates were significantly different by specific study characteristics and quality factors. Meta-regression coefficients and p-values were provided. Galbraith plot was used to display potential sources of heterogeneity. Additionally, the metareg, galbr, and metatrim macros were used for meta-analytic procedures.*

- *Forest plots:*

*Amendment(s): Forest plots were re-plotted with summary points.*

**Evaluation of bias and confounding**

*Publication bias*

- Publication bias will be assessed by inspection of the funnel plot and formal testing for

funnel plot asymmetry, using Begg’s test (Sterne JA et al. 2001).

*Amendment(s): Funnel plot in inverse-V shape was presented.*

**Discussion and Evaluating**

- The results will be critically and integratively discussed.

***Amendments in other areas:***

- *Stratification of risks by presence of a patient with TB in the household was presented across all populations.*

**References**

Higgins JP, Thompson SG, Deeks JJ, Altman DG (2003) Measuring inconsistency in meta- analysis. BMJ 327: 557-560.

Berlin JA, Longnecker MP, Greenland S (1993) Meta-analysis of epidemiologic dose- response data. Epidemiology 4: 218-228.

Sterne JA, Egger M, Smith GD (2001) Systematic review in health care: investigating and dealing with publication and other biases in meta-analysis. BMJ 323: 101-105.

Wells, G. A., Shea, B., O'Connell, D., Peterson, J., Welch, V., et al. The Newcastle-Ottawa Scale

(NOS) for assessing the quality of nonrandomized studies in meta-analysis. 2011.

http://www.ohri.ca/programs/clinical_epidemiology/oxford.asp
