# Supplementary material for: Exposure to Second-Hand Smoke and the Risk of Tuberculosis in Children and Adults: A Systematic Review and Meta-Analysis of 18 Observational Studies
Source: PLoS Med. 2015 Jun 2;12(6):e1001835. doi: 10.1371/journal.pmed.1001835 (PMC4452762; doi:10.1371/journal.pmed.1001835)
Supplement: S1 Table — (PDF) [file pmed.1001835.s002.pdf]

Supplementary Table 1: Quality assessment using Newcastle-Ottawa Scale.

Cohort studies

	Selection				Comparability	Outcome				
**Study**	Representativeness of the exposed cohort	Selection of the non exposed cohort	Ascertainment of exposure	Demonstration that the outcome of interest was not present at start of the study	Comparability of cohorts on the basis of design or analysis	Assessment of outcome	Was follow-up long enough for the outcome to occur?	Adequacy of follow up of cohorts	Total stars	Good quality
Leung et al 2010	*	*	*	*	**	*	*	*	8	Yes
Lin et al 2013	*	*	*	*	**	*	*	*	9	Yes

Cross-sectional studies†

	Selection			Comparability	Outcome			
**Study**	Representativeness of the exposed sample	Selection of the non exposed sample	Ascertainment of exposure	Comparability of outcome groups on the basis of design or analysis	Assessment of outcome	Statistical test is appropriate	Total stars	Good quality
Lindsay et al. 2014	*	*	*	**	*	*	7	Yes
Babayigit-Hocaoglu et al. 2011		*	*		*		3	No
Den Boon et al. 2007	*	*	*	**	*	*	7	Yes
Du Preez et al 2011	*	*	*	**	*	*	7	Yes
Singh et al 2005		*		*	*	*	4	No
Shin et al 2013		*	*	*	*	*	5	No

Case-control studies

	Selection				Comparability	Exposure				
**Study**	Is the case definition adequate?	Representativeness of the cases	Selection of controls	Definition of controls	Comparability of cases and controls on the basis of design or analysis	Ascertainment of exposure	Same method of ascertainment for cases and controls	Non-response rate	Total stars	Good quality
Tipayamongkholgul et al 2005	*	*		*	**	*	*	*	8	Yes
Altet 1996	*			*	**	*	*	*	7	Yes
Ariyothai 2004	*		*	*	**	*	*	*	8	Yes
Alcaide 1996	*			*	**	*	*	*	7	Yes
Patra et al 2012	*	*		*	**		*	*	7	Yes
Ramachandran et al 2011	*	*	*	*	**		*	*	8	Yes
Ozturk 2014	*			*			*	*	3	No
Gupta et al 2013	*		*	*	**		*	*	7	Yes
Garcia et al	*	*		*			*		4	No
Jubulis et al 2014	*		*	*	**		*	*	7	Yes

† We used a modified NOS scale for cross-sectional studies. Note: A score of 7 or higher was considered good study (see Supplementary Text S3 for references and original and modified scales).
