# Supplementary material for: Exposure to Second-Hand Smoke and the Risk of Tuberculosis in Children and Adults: A Systematic Review and Meta-Analysis of 18 Observational Studies
Source: PLoS Med. 2015 Jun 2;12(6):e1001835. doi: 10.1371/journal.pmed.1001835 (PMC4452762; doi:10.1371/journal.pmed.1001835)
Supplement: S2 Table — (PDF) [file pmed.1001835.s003.pdf]

Table S2. Quality assessment and subgroup analysis: second-hand smoke exposure and latent TB infection by population (with outlier excluded).

Measure or outcome	Study characteristics (No. of studies)	Summary Estimate	95% CI	I^2 (95% CI)	Meta-regression Coef. (95% CI)	p-Value
Population*	Children (5) [19-22;24]	2.00	1.30-3.08	49.5% (6.9-82.4)	(..)	(..)
Multivariate adjusted analysis	Yes (4) [20-22;24]	1.82	1.02-3.24	74.3% (45.3-88.0)	0.77 (-2.17, 3.71)	0.547
	No (1) [19]	0.81	0.33-2.01	(..)	Ref	
Adjusted for age	Yes (3) [20-22]	1.89	1.05-3.40	51.2% (0.0-83.8)	0.77 (-2.17, 3.71)	0.547
	No (2) [19;24]	2.09	1.04-4.17	70.0% (0.0-91.2)	Ref	
Adjusted for SES	Yes (3) [19;20;22]	1.36	0.77-2.39	39.1% (0.0-79.3)	-0.09 (-1.33, 1.15)	0.861
	No (2) [21;24]	2.86	2.03-4.05	0.0% (0.0-89.6)	Ref	
Adjusted for age and SES	Yes (1) [22]	1.31	0.18-2.86	(..)	-0.47 (-2.56, 1.63)	0.593
	No (4) [19-21;24]	2.09	1.31-3.34	65.1% (16.1-85.5)	Ref	
Adjusted for cooking/biomass fuel	Yes (1) [21]	2.66	1.31-5.39	(..)	-0.34 (-1.35, 2.02)	0.629
	No (4) [19;20;22;24]	1.90	1.15-3.13	64.4% (14.1-85.2	Ref	
Adjusted for BCG	Yes (1) [24]	2.93	1.97-4.36	0.0%	0.62 (-0.38, 1.62)	0.170
	No (4) [19-22]	1.61	1.00-2.74	51.1% (0.0-82.1)	Ref	
Types of study	Cross-sectional (5) [19-22;24]	2.00	1.30-3.08	49.5% (6.9-82.4)	(..)	(..)
Presence of a patient with TB in the household	Yes (4) [20-22;24]	2.79	2.02-3.84	0.0% (0.0-79.2)	0.84 (0.03, 1.65)	0.045
	No (1) [19]	1.20	0.75-1.92	(..)	Ref	
Among studies with TB contact in the household	Adjustment for TB contact (4) [20-22;24]	2.79	2.02-3.84	0.0% (0.0-79.2)	(..)	(..)
Mode of diagnosis	TST (5) [19-22;24]	2.00	1.30-3.08	49.5% (6.9-82.4)	(..)	(..)
Population**	Adults (2) [22;23]	1.58	1.03-2.43	0.0% (0.0-89.6)	(..)	(..)
Multivariate adjusted analysis	Yes (2) [22;23]	1.58	1.02-2.43	0.0% (0.0-89.6)	(..)	(..)
Adjusted for age	Yes (2) [22;23]	1.58	1.03-2.43	0.0% (0.0-89.6)	(..)	(..)
Adjusted for SES	Yes (2) [22;23]	1.58	1.03-2.43	0.0% (0.0-89.6)	(..)	(..)
Adjusted for age and SES	Yes (2) [22;23]	1.58	1.03-2.43	0.0% (0.0-89.6)	(..)	(..)
Presence of a patient with TB in the household	Yes (1) [22]	1.91	1.08-3.37	(..)	0.44 (-5.20, 6.08)	0.504
	No (1) [23]	1.23	0.64-2.37	(..)	Ref	
Mode of diagnosis	TST/QFT (2) [22;23]	1.58	1.03-2.43	0.0% (0.0-89.6)	(..)	(..)

* Pooled RRs are shown after removing an outlier[22] (US–born children). ** No studies adjusted for alcohol.
