# Supplementary material for: Exposure to Second-Hand Smoke and the Risk of Tuberculosis in Children and Adults: A Systematic Review and Meta-Analysis of 18 Observational Studies
Source: PLoS Med. 2015 Jun 2;12(6):e1001835. doi: 10.1371/journal.pmed.1001835 (PMC4452762; doi:10.1371/journal.pmed.1001835)
Supplement: S3 Table — (PDF) [file pmed.1001835.s004.pdf]

Table S3. Quality assessment and subgroup analysis: second-hand smoke exposure and active TB disease by population.

Measure or outcome	Study characteristics (No. of studies)	Summary Estimate	95% CI	I^2 (95% CI)	Meta-regression Coef. (95% CI)	p -Value
**Population**	**Children (7) [18;26;29;31;33-35]**	**3.41**	**1.81-6.45**	**72.1% (39.6-87.1)**	**(..)**	**(..)**
Outcome	Exclusive pulmonary TB (4) [18;26;29;31]	2.96	1.11-7.85	68.4% (8.5-89.1)	0.34 (-1.48, 2.17)	0.649
	Pulmonary or extra-pulmonary TB (3) [33-35]	4.29	1.35-13.65	83.3% (49.5-94.5)	Ref	
Quality of study	Good (6) [26;29;31;33-35]	4.33	2.13 (8.79)	68.6% (26.0-86.7)	1.26 (-0.69, 3.20)	0.157
	Poor to moderate (1) [18]	1.20	0.63-2.30	0.0%	Ref	
Multivariate adjusted analysis	Yes (6) [26;29;31;33-35]	4.33	2.13 (8.79)	68.6% (26.0-86.7)	1.26 (-0.69, 3.21)	0.157
	No (1) [18]	1.20	0.63-2.30	(..)	Ref	
Adjusted for age	Yes (6) [26;29;31;33-35]	4.33	2.13 (8.79)	68.6% (26.0-86.7)	1.26 (-0.69, 3.21)	0.157
	No (1) [18]	1.20	0.63-2.30	(..)	Ref	
Adjusted for SES	Yes (5) [26;29;31;33;34]	3.64	1.78-7.46	63.6% (4.1-86.2)	0.25 (-1.73, 2.22)	0.762
	No (2) [18;35]	3.19	0.43-23.67	90.1%	Ref	
Adjusted for age and SES	Yes (4) [26;29;31;34]	5.29	3.03-9.22	0.0% (0.0-84.7)	0.82 (-0.69, 3.33)	0.221
	No (3) [18;33;35]	2.36	0.98-5.68	80.6% (39.1-93.8)	Ref	
Adjusted for cooking/biomass fuel	Yes (5) [18;29;33-35]	2.91	1.41-5.98	74.6% (37.2-89.7)	-0.73 (-2.78, 1.32)	0.405
	No (2) [26;31]	5.64	2.65-11.96	0.0%	Ref	
Adjusted for BCG	Yes (1) [26]	5.39	2.44-11.91	(..)	0.53 (-1.86, 2.92)	0.593
	No (6) [[18;29;31;33-35]	3.11	1.55-6.24	70.6% (31.5-87.4)	Ref	
Types of study	Cohort (1) [31]	8.48	0.77-93.48	(..)	0.96 (-2.79, 4.70)	0.541
	Case-control (6) [18;26;29;33-35]	3.25	1.68-6.28	75.6% (44.9-89.2)	Ref	
Type of control among case-control studies	Community based (2) [29;34]	4.90	2.15-11.17	0.0%	-0.18 (-3.85, 3.49)	0.886
	Hospital based (3) [18;33;35]	2.36	0.98-5.68	80.6% (39.1-93.8)	-0.81 (-4.10, 2.49)	0.493
	Close contacts of cases (1) [26]	5.39	2.44-11.91	(..)	Ref	
Presence of a patient with TB in the household	Yes (5) [26;29;33-35]	4.14	1.96-8.75	73.6% (34.2-89.4)	0.81 (-1.07, 2.69)	0.319
	No (2) [18;31]	2.24	0.38-13.33	57.9%	Ref	
Among studies with TB contact in the household	Adjustment for TB contact (2) [29;33]	1.79	1.20-2.66	0.0%	-1.28 (-2.37, -0.19)	0.033
	No adjustment for TB contact (3) 26;34;35]	6.45	3.76-11.05	0.0% (0.0-89.6)	Ref	
Mode of diagnosis	microbiological (3) [18;26;29]	2.57	0.88-7.49	76.1% (21.5-92.7)	-.71 (-3.58, 5.00)	0.670
	Radiographic findings/TST (1) [31]	8.48	0.77-93.48	(..)	-0.49 (-2.55, 1.58)	0.550
	Others (3) [33-35]	4.29	1.35-13.65	83.3% (49.5-94.5)	Ref	
**Population**	**Adults (6) [25;27;28;30-32]**	**1.32**	**1.04-1.68**	**42.3% (0.0-74.5)**	**(..)**	**(..)**
Outcome	Pulmonary TB only (5) [25;27;28;31;32]	1.23	0.91-1.66	48.0% (0.0-78.0)	0.14 (-1.1, 1.39)	0.787
	Pulmonary or extra-pulmonary TB (1) [30]	1.49	1.01-2.19	(..)	Ref	
Quality of study	Good (5) [25;27;28;30;31]	1.34	1.03-1.74	50.0% (0.0-78.8)	0.08 (-1.29, 1.44)	0.896
	Poor to moderate (1) [32]	1.25	0.69-2.25	(..)	Ref	
Multivariate adjusted analysis	Yes (5) [25;27;28;30;31]	1.34	1.03-1.74	50.0% (0.0-78.8)	0.08 (-1.29, 1.44)	0.896
	No (1) [32]	1.25	0.69-2.25	(..)	Ref	
Adjusted for age	Yes (5) [25;27;28;30;31]	1.34	1.03-1.74	50.0% (0.0-78.8)	0.08 (-1.29, 1.44)	0.896
	No (1) [32]	1.25	0.69-2.25	(..)	Ref	
Adjusted for alcohol	Yes (2) [30;31]	1.26	0.94-1.70	49.1% (0.0-83.2)	-0.16 (-1.14, 0.82)	0.702
	No (4) [25;27;28;32]	1.45	0.79-2.66	49.7% (0.0-83.4)	Ref	
Adjusted for SES	Yes (4) [25;28;30;31]	1.24	0.79-1.95	52.4% (0.0-81.0)	-0.25 (-1.35, 0.85)	0.595
	No (2) [27;32]	1.50	0.91-2.48	23.7%	Ref	
Adjusted for age and SES	Yes (4) [25;27;28;31]	1.32	0.76-2.30	56.6% (0.0-82.5)	-0.06 (-1.10, 0.98)	0.897
	No (2) [30;32]	1.41	1.02-1.95	0.0%	Ref	
Adjusted for cooking/biomass fuel	Yes (2) [28;32]	1.03	0.61-1.73	45.9%	-0.46 (-1.55, 0.62)	0.339
	No (4) [25;27;30;31]	1.41	1.08-1.85	45.3% (0.0-78.4)	Ref	(..)
Adjusted for BCG	Yes (1) [27]	2.37	0.94-5.99	(..)	0.65 (-0.88, 2.17)	0.340
	No (5) [25;28;30-32]	1.27	0.99-1.62	42.9% (0.0-76.0)	Ref	
Types of study	Cohort (2) [30;31]	1.26	0.94-1.70	49.1% (0.0-83.2)	-0.16 (-1.14, 0.82)	0.702
	Case-control (4) [25;27;28;32]	1.44	0.96-2.17	49.7% (0.0-83.4)	Ref	
Type of control among case-control studies	Hospital based (2) [27;32]	1.55	0.85-2.82	23.7%	0.31 (-3.22, 3.85)	0.738
	Close contacts of cases (2) [25;28]	1.18	0.25-5.48	78.2%	Ref	
Presence of a patient with TB in the household	Yes (2) [27;30]	1.60	1.12-2.28	0.0%	0.38 (-0.59, 1.36)	0.374
	No (4) [25;28;31;32]	1.13	0.82-1.56	46.6% 0.0-78.9)	Ref	
Among studies with TB contact in the household	Adjustment for TB contact (1) [27]	2.37	0.94-5.99	(..)	(..)	(..)
	No adjustment for TB contact (1) [30]	1.49	1.01-2.19	(..)	(..)	(..)
Mode of diagnosis	microbiological (5) [25;27;28;30;32]	1.47	1.11-1.94	33.1% (0.0-74.6)	-0.32 (-1.28, 0.65)	0.452
	Radiographic findings/TST (1) [31]	1.13	0.56-2.27	52.7% (0.0-86.4)	Ref	
