# Supplementary material for: Exposure to Second-Hand Smoke and the Risk of Tuberculosis in Children and Adults: A Systematic Review and Meta-Analysis of 18 Observational Studies
Source: PLoS Med. 2015 Jun 2;12(6):e1001835. doi: 10.1371/journal.pmed.1001835 (PMC4452762; doi:10.1371/journal.pmed.1001835)
Supplement: S2 Text — (DOC) [file pmed.1001835.s006.doc]

**NEWCASTLE - OTTAWA QUALITY ASSESSMENT SCALE**

**CASE CONTROL STUDIES**

Note: A study can be awarded a maximum of one star for each numbered item within the Selection and Exposure categories. A maximum of two stars can be given for Comparability.

**Selection**

1) Is the case definition adequate?

a) yes, with independent validation **🟑**

b) yes, eg record linkage or based on self reports

c) no description

2) Representativeness of the cases

a) consecutive or obviously representative series of cases **🟑**

b) potential for selection biases or not stated

3) Selection of Controls

a) community controls **🟑**

b) hospital controls

c) no description

4) Definition of Controls

a) no history of disease (endpoint) **🟑**

b) no description of source

**Comparability**

1) Comparability of cases and controls on the basis of the design or analysis

a) study controls for _______________ (Select the most important factor.) **🟑**

b) study controls for any additional factor **🟑** (This criteria could be modified to indicate specific control for a second important factor.)

**Exposure**

1) Ascertainment of exposure

a) secure record (eg surgical records) **🟑**

b) structured interview where blind to case/control status **🟑**

c) interview not blinded to case/control status

d) written self report or medical record only

e) no description

2) Same method of ascertainment for cases and controls

a) yes **🟑**

b) no

3) Non-Response rate

a) same rate for both groups **🟑**

b) non respondents described

c) rate different and no designation

**NEWCASTLE - OTTAWA QUALITY ASSESSMENT SCALE**

**COHORT STUDIES**

Note: A study can be awarded a maximum of one star for each numbered item within the Selection and Outcome categories. A maximum of two stars can be given for Comparability

**Selection**

1) Representativeness of the exposed cohort

a) truly representative of the average _______________ (describe) in the community **🟑**

b) somewhat representative of the average ______________ in the community **🟑**

c) selected group of users eg nurses, volunteers

d) no description of the derivation of the cohort

2) Selection of the non exposed cohort

a) drawn from the same community as the exposed cohort **🟑**

b) drawn from a different source

c) no description of the derivation of the non exposed cohort

3) Ascertainment of exposure

a) secure record (eg surgical records) **🟑**

b) structured interview **🟑**

c) written self report

d) no description

4) Demonstration that outcome of interest was not present at start of study

a) yes **🟑**

b) no

**Comparability**

1) Comparability of cohorts on the basis of the design or analysis

a) study controls for _____________ (select the most important factor) **🟑**

b) study controls for any additional factor **🟑** (This criteria could be modified to indicate specific control for a second important factor.)

**Outcome**

1) Assessment of outcome

a) independent blind assessment **🟑**

b) record linkage **🟑**

c) self report

d) no description

2) Was follow-up long enough for outcomes to occur

a) yes (select an adequate follow up period for outcome of interest) **🟑**

b) no

3) Adequacy of follow up of cohorts

a) complete follow up - all subjects accounted for **🟑**

b) subjects lost to follow up unlikely to introduce bias - small number lost - > ____ % (select an adequate %) follow up, or description provided of those lost) **🟑**

c) follow up rate < ____% (select an adequate %) and no description of those lost

d) no statement

**NEWCASTLE - OTTAWA QUALITY ASSESSMENT SCALE**

**(adapted for cross-sectional studies)**

**Selection:** (Maximum 3 stars)

1) Representativeness of the sample:

a) Truly representative of the average in the target population. **🟑** (all subjects or random sampling)

b) Somewhat representative of the average in the target population. **🟑** (non-random sampling)

c) Selected group of users.

d) No description of the sampling strategy.

2) Non-respondents:

a) Comparability between respondents and non-respondents characteristics is established, and the response rate is satisfactory. **🟑**

b) The response rate is unsatisfactory, or the comparability between respondents and non-respondents is unsatisfactory.

c) No description of the response rate or the characteristics of the responders and the non-responders.

3) Ascertainment of the exposure (risk factor):

a) Validated measurement tool. **🟑**

b) Non-validated measurement tool, but the tool is available or described.

c) No description of the measurement tool.

**Comparability:** (Maximum 2 stars)

1) The subjects in different outcome groups are comparable, based on the study design or analysis. Confounding factors are controlled.

a) The study controls for the most important factor (select one). **🟑**

b) The study control for any additional factor. **🟑**

**Outcome:** (Maximum 2 stars)

1) Assessment of the outcome:

a) Independent blind assessment. **🟑**

b) Record linkage. **🟑**

c) Self report.

d) No description.

2) Statistical test:

a) The statistical test used to analyze the data is clearly described and appropriate, and the

measurement of the association is presented, including confidence intervals and the probability level (p value). **🟑**

b) The statistical test is not appropriate, not described or incomplete.

This scale has been adapted from the Newcastle-Ottawa Quality Assessment Scale for cohort and case-control studies to perform a quality assessment of cross-sectional studies for the systematic review, “Exposure to second-hand smoke and the risk of tuberculosis in children and adults: systematic review and a meta-analysis of 18 observational studies”. This scale was a modified version of the NOS scale, as also used by several other studies that have felt the need to adapt the NOS scale so as to appropriately assess the quality of cross-sectional studies.

We did a comprehensive search on literature and found that a NOS score of 7 or more can be considered a “good” study (see McPheeters et al. 2012; see Appendix G page 103-104 in <http://www.ncbi.nlm.nih.gov/pubmedhealth/PMH0049229/>). So we used this criterion as a cut off for good quality study.

**References**

Wells, G. A., Shea, B., O'Connell, D., Peterson, J., Welch, V., et al. The Newcastle-Ottawa Scale (NOS) for assessing the quality of nonrandomized studies in meta-analysis. 2011. <http://www.ohri.ca/programs/clinical_epidemiology/oxford.asp>

McPheeters, M.L., Kripalini, S., Peterson, N.B., Idowu, R.T. et al. (2012). Quality Improvement Interventions To Address Health Disparities. Evidence Report/ technology Assessment. Rockville (MD): Agency for Healthcare Research and Quality (US). <http://www.ncbi.nlm.nih.gov/pubmedhealth/PMH0049222/pdf/TOC.pdf>
